# Boosting planar perovskite solar cell performance *via* peripheral end-group engineering of phenoxazine-core hole transport materials

**DOI:** 10.1039/d5sc04399a

**Published:** 2025-09-10

**Authors:** Murali Ravi, Ziyang Xia, Divya Kumar, Cheng Chen, Haoxin Wang, Yi Tian, Balamurali Ravichandran, Ming Cheng

**Affiliations:** a Institute for Energy Research, School of Materials Science and Engineering, Jiangsu University Zhenjiang 212013 China chencheng@ujs.edu.cn mingcheng@ujs.edu.cn

## Abstract

Developing novel, cost-effective hole-transporting materials (HTMs) with tailored chemical/electronic properties is critical for high-performance, stable planar perovskite solar cells (PSCs). Herein, we design three HTMs based on a di-trifluoromethyl-substituted phenoxazine core linked to distinct peripheral end groups: pcz-SM-DM, pcz-DM, and pcz-SM. The polyfluorinated core effectively passivates interfacial defects, while peripheral dimethyl fluorene units modulate HTM energy levels and charge carrier mobilities. Among these reported HTMs, pcz-SM exhibits the most suitable energy alignment and enhanced hole transport. Devices using pcz-SM as the HTM achieve an exceptional power conversion efficiency (PCE) of 25.3% and maintain 81% initial stability after 1000 hours of ambient aging. These results validate that tailoring peripheral end groups for this novel core is a powerful molecular engineering strategy to boost charge carrier mobility and photovoltaic performance in PSCs.

## Abstract 2

A cost-effective di-trifluoromethyl-substituted phenoxazine core based hole transporting material (HTM) is reported, achieving an impressive efficiency of 25.3%

## Graphical abstract

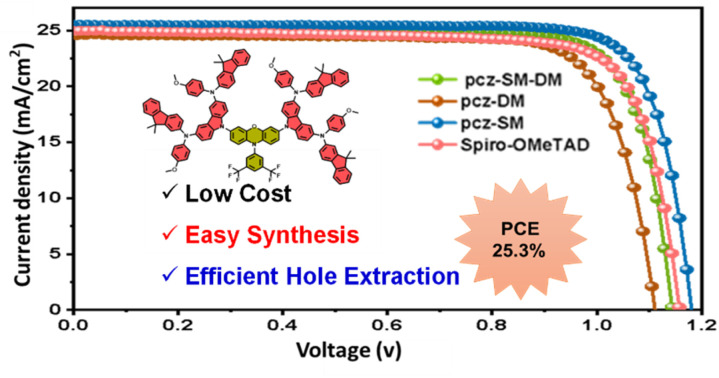

Most recently, power conversion efficiency (PCE) of organic–inorganic hybrid perovskite solar cells (PSCs) has soared from a modest 3.8% to a remarkable 27.0%, making them a promising alternative to conventional silicon-based solar cells.^1–5^ The future development of PSCs will focus on efficiency enhancement, stability improvement, and cost reduction. Advancements in these areas will pave the way toward large-scale commercial adoption and widespread utilization of PSCs.^6–9^ The hole transport material (HTM) is recognized as a crucial component of PSCs and is responsible for hole extraction/transporting, preventing unwanted charge recombination, shielding the active layer from harmful external elements like moisture, and metal migration from the electrodes. As a good option for attaining highly efficient PSCs, small molecular HTMs with well-aligned energy levels provide a significant advantage for efficient charge transport.^10–12^ To date, 2,2′,7,7′-tetrakis[*N*,*N*-di(4-methoxyphenyl)amino]-9,9-spirobifluorene (Spiro-OMeTAD) is the most prominent and widely used HTM in PSCs and is used as a benchmark to evaluate the performance of newly developed HTMs.^13–15^ However, the high cost of Spiro-OMeTAD due to the complex synthesis and purification process limits its large-scale application in future PSC production.^16,17^ These obstacles drove researchers to develop low-cost alternative small molecular HTMs. Moreover, the significant energy gap between the highest occupied molecular orbital (HOMO) of Spiro-OMeTAD and the valence band (VB) of the perovskite material hinders efficient hole extraction and transport, resulting in substantial losses in open-circuit voltage (*V*_OC_), which negatively impacts overall device performance.^18,19^ Furthermore, surface defects, such as ion vacancies and uncoordinated species, which are usually present at the grain boundaries and polycrystalline perovskite film surface, are reported to be nonradiative recombination centres and accelerate the degradation of PSCs. Thus, besides the synthesis cost consideration, it is also highly desirable to endow HTMs with excellent hole transport ability, tunable energy levels, and a defect passivation function to synergistically enhance the stability and PCE of PSCs. Phenoxazine (POZ) boasts a conjugated electron-rich structure with a nitrogen-containing heterocyclic core and a unique butterfly configuration, which can be readily modified through a straightforward synthetic process, ensuring low material cost and tunable HOMO energy levels.^20–22^ In addition, POZ-based molecular materials exhibit exceptional charge transport, photo-conductive and photo-refractive properties, making them popular choices for various optoelectronic applications.^23–26^ While phenoxazine-based HTMs haven't been fully explored for application in PSCs, through precise molecular structure design and regulation, impressive photovoltaic performance could be expected.

Based on the above concerns, we report three simple and affordable POZ-based small molecular HTMs termed pcz-SM-DM, pcz-DM, and pcz-SM. By introducing an electron-deficient 1,3-bis(trifluoromethyl)-benzene group through *N*-substitution, we successfully weakened the electron-donating ability of the nitrogen atom in the POZ ring, thereby lowering the HOMO level of these HTMs. This design strategy also offers POZ-based HTMs interaction sites to passivate surface defects of the perovskite film. Different types of peripheral end groups are tailored to further tune the geometrical structure, energy level and electronic properties of HTMs, which are crucial parameters to evaluate HTMs' properties. The designed HTMs, pcz-SM-DM, pcz-DM and pcz-SM, can be efficiently synthesized through a palladium-catalyzed cross-coupling reaction (Scheme 1 and Fig. S1–S15) with total yields of 66.9%, 74.0% and 78.1%, respectively. The synthesis costs of pcz-SM-DM, pcz-DM, and pcz-SM are calculated to be $70.0 g^−1^, $36.2 g^−1^, and $32.0 g^−1^, respectively, which are significantly lower than that of Spiro-OMeTAD ($200 g^−1^).^27,28^ This straightforward synthetic approach highlights the accessibility of these materials, making them promising and cost-effective candidates for further investigation. The incorporation of carbazole linked to the *N*-(4-methoxyphenyl)-9,9-dimethyl-9*H*-fluorene group onto the phenoxazine core was performed to enhance the target materials solubility, hole mobility, conductivity, and spatial configuration.^29^

**Scheme 1:**
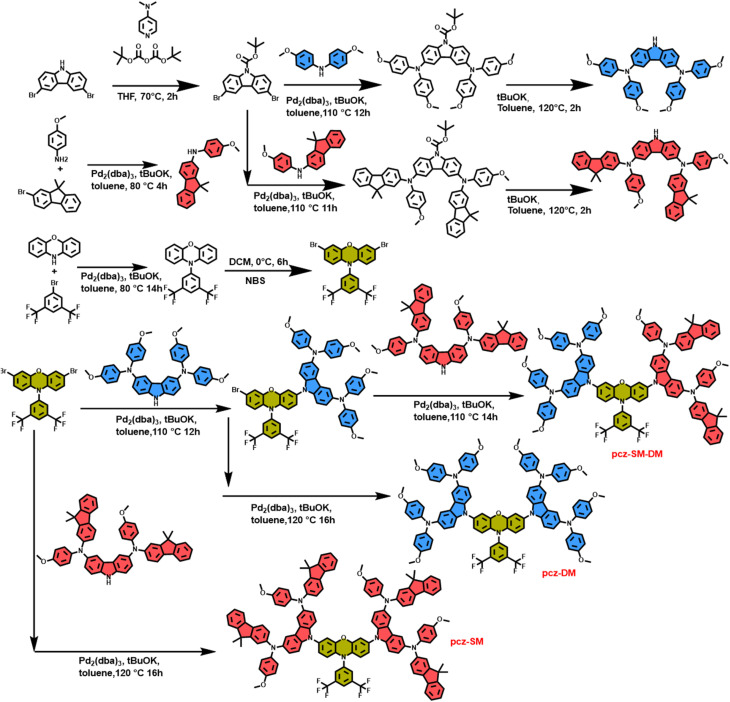
Synthesis routes of pcz-SM-DM, pcz-DM, and pcz-SM.

As the number of fluorene groups increases, the properties of HTMs gradually improve. The pcz-SM-based PSC achieved a remarkable PCE of 25.3%, paired with outstanding long-term stability, retaining more than 80% of its original PCE even after aging for 1000 hours under ambient conditions (30–50% relative humidity), showing its potential for durable and high-efficiency energy conversion.

The thermal analysis reveals significant differences in stability among the three HTMs, in that pcz-SM demonstrates superior performance. The Thermo-gravimetric Analysis (TGA) and Differential Scanning Calorimetry (DSC) results are shown in Fig. S16. The TGA results show that pcz-SM possesses the highest (Td – with 5% weight loss) decomposition temperature (440 °C), followed by pcz-SM-DM (422 °C) and pcz-DM (376 °C), establishing a clear thermal stability trend: pcz-SM > pcz-SM-DM > pcz-DM. This hierarchy suggests that peripheral dimethyl fluorene functionalization promotes optimal molecular packing and thermal resilience, while asymmetrical pcz-SM-DM introduces structural compromises, and pcz-DM provides the weakest stabilization. The DSC results further support this trend, with pcz-SM exhibiting the highest glass transition temperature (*T*_g_) (184 °C), compared to pcz-SM-DM (162 °C) and pcz-DM (147 °C), indicating stronger crystalline ordering in pcz-SM, which is significantly higher than that of Spiro-OMeTAD (121 °C).^30–32^ These findings highlight that dimethyl fluorene end group modified pcz-SM yields more thermally robust HTMs and the most promising candidates for device implementations where thermal stability is paramount, while pcz-SM-DM may offer intermediate properties for specific processing conditions. As shown in Fig. 1a, all HTMs exhibit prominent absorption peaks in the UV region. Strong absorption bands appear at 352 nm (pcz-SM-DM), 307 nm (pcz-DM), and 352 nm (pcz-SM) which are attributed to intramolecular charge transfer (ICT) from the peripheral end groups to the core unit. HTMs pcz-SM-DM and pcz-SM exhibit a pronounced red shift of 45 nm compared to pcz-DM due to the introduction of dimethyl-fluorene groups with a larger conjunction. This extended conjugation length facilitates more efficient intermolecular accumulation.^33^ The intermolecular accumulation of pcz-SM-DM, pcz-DM and pcz-SM materials was further substantiated through UV-Vis spectroscopy using a range of polar solvents (DMF, ethyl acetate, THF, and dioxane). As illustrated in Fig. S17a–c, the absorption wavelengths of these materials exhibited a pronounced shift in response to varying solvent polarities. Notably, increasing solvent polarity led to a redshift in the absorption wavelengths of pcz-SM-DM and pcz-SM, whereas pcz-DM displayed minimal wavelength shifts under the same conditions. The optical band gaps (*E*_g_) are calculated to be 2.78, 2.87 and 2.86 eV for HTMs pcz-SM-DM, pcz-DM and pcz-SM, respectively. According to cyclic voltammograms (CVs) of the HTMs (Fig. 1b), all materials show reversible redox peaks, indicating strong electrochemical stability of the reported HTMs. The HOMO energy levels are determined to be −5.10, −5.08 and −5.12 eV for HTMs pcz-SM-DM, pcz-DM and pcz-SM, respectively, which are all comparable to that of Spiro-OMeTAD (−5.07 eV) (see Fig. S17d). The values are more positive than the VB of the mixed perovskite (−5.65 eV) ((FAPbI_3_)_0.92_(MAPbBr_3_)_0.08_),^28^ indicating that hole extraction is feasible from the energy level alignment. The lowest unoccupied molecular orbital (LUMO) energy levels of reported HTMs are high enough (see Table 1) for blocking the back-transfer of electrons.^34,35^ Ultraviolet photoelectron spectroscopy (UPS) measurements of the HTMs in their pristine film state revealed HOMO energies of −5.11 eV (pcz-SM), −5.09 eV (pcz-SM-DM), −5.03 eV (pcz-DM), and −5.04 eV (Spiro-OMeTAD), as shown in Fig. 1d. This established a consistent energetic trend among the series, which mirrored the ordering previously observed in CV. Given that operational devices employ doped HTMs, further UPS analysis was conducted on films incorporating the common additives lithium bis(trifluoromethanesulfonyl)imide (Li-TFSI) and 4-*tert*-butylpyridine (TBP). The introduction of these dopants induced a significant shift to deeper HOMO levels (Fig. 1e). The doped HOMO energies were measured at −5.21, −5.17, −5.12, and −5.18 eV for pcz-SM, pcz-SM-DM, pcz-DM, and Spiro-OMeTAD, respectively. This downward shift in energy creates optimal alignment with the valence band of typical perovskite materials, ensuring efficient hole extraction and transport. Density Functional Theory (DFT) calculations were performed to investigate the charge distribution in pcz-SM-DM, pcz-DM, and pcz-SM, providing valuable insights into their electronic structures (Fig. 1f). Notably, the HOMO is delocalized along the backbone in all HTMs, while the LUMO is localized on the *N*-substituent 3,5-bis(trifluoromethyl)benzene group. This spatial separation of HOMO/LUMO levels enhances hole extraction and transport.^36^ DFT calculations revealed the following HOMO and LUMO energy levels for pcz-SM-DM: −4.25/−1.79 eV, pcz-DM: −4.24/−1.78 eV, and pcz-SM: −4.39/−1.80 eV, respectively (Fig. 1d). The additional dimethyl fluorene group in pcz-SM lowers its HOMO energy level, making it more favourable than pcz-DM and pcz-SM-DM, consistent with the CV trend. The ESP results show that the number of dimethyl-fluorene substitutions has a minimal impact on the surface potential of peripheral end groups. In conclusion, all HTMs exhibit promising properties for efficient hole extraction in PSCs, facilitating efficient charge transport and enhancing photoelectronic conversion. Furthermore, reorganization energies (*λ*) are calculated (Fig. 1g). They provide critical insight into the hole transfer efficiency of new HTMs. The values were found to be 0.60 eV for pcz-SM-DM, 0.70 eV for pcz-DM, 0.15 eV for pcz-SM, and 0.49 eV for Spiro-OMeTAD. Notably, pcz-SM exhibits a remarkably low *λ* of 0.15 eV, which is substantially lower than that of the other materials. A small *λ* implies minimal geometric rearrangement between the neutral and ionic states, which is a hallmark of a structurally rigid molecule. This facilitates faster and more efficient charge hopping between molecules, a prerequisite for high hole mobility. In contrast, the higher *λ* values for pcz-SM-DM and pcz-DM suggest greater molecular flexibility upon oxidation, which could hinder charge transport.^37,38^

**Fig. 1:**
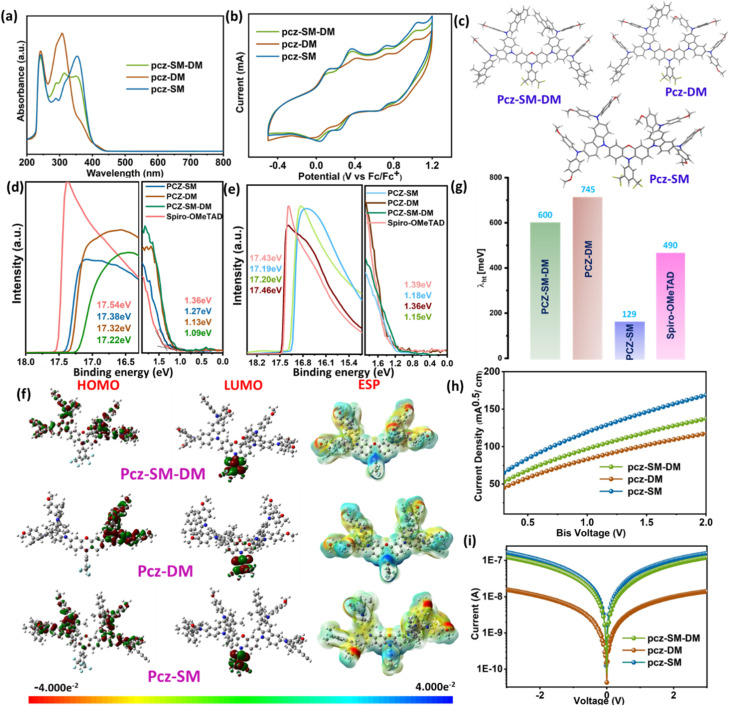
(a) UV absorption spectra in THF (2 × 10^−5^ mol L^−1^); (b) CV curve analyses in DCM (1.0 × 10^−3^M); (c) the optimized structures of HTMs; UPS spectra of (d–e) un-doped and doped HTMs in a solid thin film; (f) DFT spatial distribution of HOMO and LUMO levels and ESP; (g) reorganization energy (*λ*_ht_) associated with hole transport, (h) hole mobility, and (i) conductivity of pcz-SM-DM, pcz-DM, and pcz-SM, HTMs.

**Table 1:** Photophysical and electrochemical properties of pcz-DM, pcz-SM-DM, and pcz-SM

HTM	*E*_HOMO_ (eV)	*E*_0−0_ (eV)	*E*_LUMO_ (eV)	Conductivity (S cm^−1^)	*μ*_h_ (cm^2^/(Vs))
pcz-SM-DM	−5.10	2.78	−2.31	7.86 × 10^−4^	4.93 × 10^−4^
pcz-DM	−5.08	2.87	−2.23	9.80 × 10^−5^	3.57 × 10^−4^
pcz-SM	−5.12	2.86	−2.26	10.3 × 10^−4^	7.75 × 10^−4^
Spiro-OMeTAD	−5.07	—	—	8.91 × 10^−4^	5.32 × 10^−4^

The hole mobility and conductivity of these three HTMs are in the same magnitude as those of Spiro-OMeTAD, and the HTM pcz-SM with extended conjunction exhibited the highest hole mobility of 7.75× 10^−4^ cm^2^ V^−1^ S^−1^ (Fig. 1h and S17e) and conductivity of 10.03 × 10 ^−3^ S cm^−1^ (Fig. 1i and S17f), which is beneficial for enhancing the hole extraction of holes and improving the fill factor (FF) of PSCs. Overall, the polyfluorinated core and dimethyl-fluorene groups enhance the energy level alignment, hole mobility, and conductivity, suggesting improved performance in the final device.^39,40^

The SEM images shown in Fig. 2a reveal that all HTMs can form a uniform film and completely cover the perovskite layer, which enables excellent contact between the hole transport and perovskite layers, leading to efficient charge transport. With the deposition of HTMs on the perovskite film, the root mean square (RMS) roughness of the films obviously reduced. The RMS values are determined to be 11.0, 14.3, 10.7 and 12.8 nm for pcz-SM-DM, pcz-DM, pcz-SM and Spiro-OMeTAD based samples (Fig. 2b), respectively. This reduced roughness can improve the interface contact, in turn, minimize non-radiative charge recombination and result in better performance. Moreover, the hydrophobicity is enhanced with the introduction of developed HTMs (Fig. 2c), which is ascribed to increased surface homogeneity and fluoric functional groups on the core moiety.^41,42^ The improved hydrophobicity is beneficial for protecting the underlying perovskite layer from moisture and improving the device's long-term humidity stability.

**Fig. 2:**
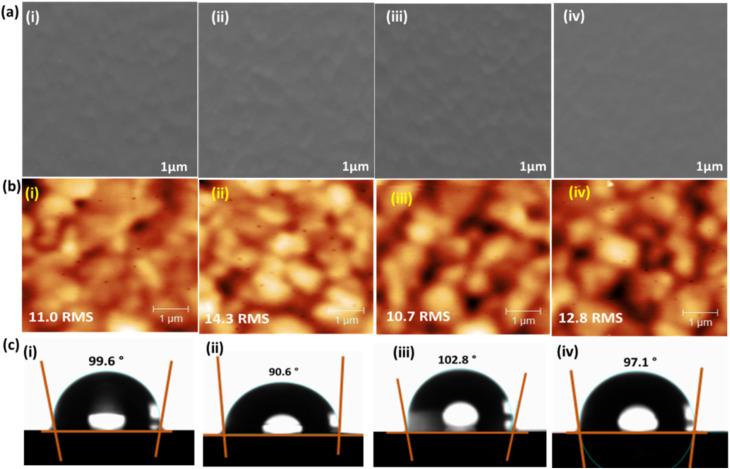
(a) The top-view of SEM, (b) AFM, and (c) water contact angle images of (i) pcz-SM-DM, (ii) pcz-DM, (iii) pcz-SM, and (iv) Spiro-OMeTAD based devices.

The photovoltaic properties of pcz-SM-DM, pcz-DM, pcz-SM and Spiro-OMeTAD are evaluated with the device structure of FTO/SnO_2_/perovskite/HTM/Au following our previous method.^43^ As shown in Fig. 3a and Table 2, the devices based on pcz-SM-DM, pcz-DM, and pcz-SM achieved an impressive PCE of 24.0%, 21.7%, and 25.3%, respectively. Notably, the pcz-SM-based device outperformed the reference device (HTM Spiro-OMeTAD −24.4%), reaching a champion PCE of 25.3%. According to the incident photon-to-current conversion efficiency (IPCE) spectra (Fig. 3b), all devices exhibit wide light responses between 380 and 780 nm, with IPCE values exceeding 85%. The integrated *J*_SC_ values are 24.1, 23.6, 24.7, and 24.3 mA cm^−2^ for pcz-SM-DM, pcz-DM, pcz-SM, and Spiro-OMeTAD, respectively, which closely match the *J*_SC_ values obtained from the *J*–*V* curves.

**Fig. 3:**
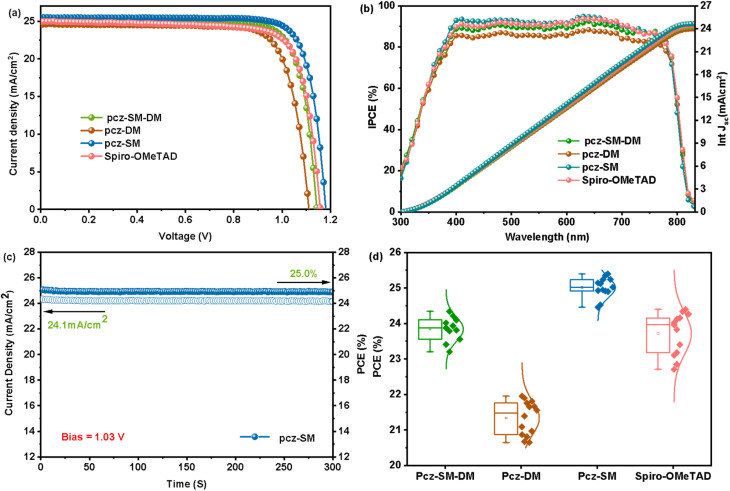
(a) *J*–*V* curves, (b) IPCE curves and *J*_SC_ integration of PSCs containing pcz-SM-DM, pcz-DM, pcz-SM, and Spiro-OMeTAD as HTM. (c) Steady-state PCE of PSCs containing pcz-SM as the HTM at the MPP point, and (d) reproducibility of PSCs based on different HTMs.

**Table 2:** The champion device parameters (forward scan) for n–i–p structured PSCs at pcz-SM-DM, pcz-DM, pcz-SM, and Spiro-OMeTAD as HTLs

HTM	*V*_OC_ (V)	*J*_SC_ (mA cm^−2^)	FF (%)	PCE (%)
pcz-SM-DM	1.15	24.9	83.7	24.0
pcz-DM	1.11	24.6	79.6	21.7
pcz-SM	1.18	25.5	84.2	25.3
Spiro-OMeTAD	1.16	25.3	83.1	24.4

Notably, the IPCE values for pcz-SM-based devices are slightly higher than those for Spiro-OMeTAD-based devices, indicating more efficient photoelectric conversion. To validate the device performance obtained from the *J*–*V* curves, we measured the steady power output (SPO) for 300 seconds for devices based on pcz-SM-DM, pcz-DM, pcz-SM, and Spiro-OMeTAD (with bias values of 0.99, 0.91, 1.03, and 1.01 V, respectively). The results show that all devices exhibit stable PCE outputs, with values of 23.6%, 21.2%, 25.0%, and 24.1%, respectively (Fig. 3c and S18a–c). Additionally, the stabilized *J*_SC_ values are 23.7, 23.4, 24.1, and 23.8 mA cm^−2^, respectively. The PSCs based on pcz-SM-DM, pcz-DM, pcz-SM, and Spiro-OMeTAD show narrow distributions of parameters of PCE, *V*_OC_, *J*_SC_, and FF, indicating good reproducibility across 20 devices (Fig. 3d and S19). The average PCEs are 23.8% ± 0.34, 21.4% ± 0.47, 25.0% ± 0.28, and 23.7% ± 0.59, respectively. The superior performance of pcz-SM-based PSCs compared to Spiro-OMeTAD can be attributed to better film formation properties, interfacial contact with perovskite, and more efficient hole extraction and transport capabilities, ultimately leading to higher *V*_OC_ and *J*_SC_.

This conclusion is further supported by the results of steady-state and time-resolved photoluminescence (PL) decay measurements, which provide additional evidence for the superior charge extraction and transport properties of pcz-SM. The fluorescence intensity of the perovskite film is quenched with the introduction of HTMs due to hole transport from the perovskite to the HTMs (Fig. 4a). Notably, perovskite/pcz-SM exhibits higher hole extraction efficiency and quenching rates compared to pcz-SM-DM, pcz-DM and Spiro-OMeTAD, indicating more efficient hole transport in pcz-SM-based PSCs, which is consistent with its higher PCE. All new HTMs show a blue-shifted fluorescence peak relative to Spiro-OMeTAD, indicating effective surface defect passivation due to the fluorine group.^44–46^ To further investigate this phenomenon, X-ray Photoelectron Spectroscopy (XPS) analysis was performed. As depicted in Fig. 4b, a distinct peak at a binding energy of 687.4 eV is observed, corresponding to the F 1s peak of pcz-SM-DM, pcz-DM, and pcz-SM. This finding unequivocally confirms the presence of fluorine on the surface of the perovskite material.^47^ In the Pb 4f XPS spectra shown in Fig. 4c, the pristine perovskite film displays two additional peaks at 136.6 and 141.5 eV, attributed to metallic Pb resulting from iodine and cation vacancies. The presence of metallic Pb signifies surface defects, which can compromise the performance and stability of PSCs. The Pb 4f peaks (4f_7/2_ and 4f_5/2_) show a significant +0.35 eV binding energy shift when coated with our designed HTMs (pcz-SM-DM, pcz-DM, and pcz-SM), indicating strong coordination between these HTMs and Pb^2+^ ions at the interface. In contrast, perovskite coated with conventional HTM Spiro-OMeTAD exhibits a much smaller shift (<0.1 eV), demonstrating substantially weaker interaction with the Pb^2+^ sites. These results clearly demonstrate that our custom-designed HTMs form more robust interfacial coordination with the perovskite compared to standard Spiro-OMeTAD.^30,48^ The TRPL measurements (Fig. 4d) and Table S10 reveal two distinct decay processes: a fast initial decay (*τ*_1_) attributed to carrier extraction to the HTMs and a slower decay (*τ*_2_) attributed to interface recombination losses.^49^ With the increasing number of dimethyl-fluorine end groups, the charge carrier lifetime (*τ*) of perovskite/HTMs decreases. The perovskite/pcz-SM sample shows the shortest charge carrier lifetime, indicating the fastest hole extraction rate. This is attributed to higher hole mobility, conductivity, and suitable energy level alignment with the perovskite of HTM pcz-SM.^50^ As a result, pcz-SM-based devices exhibit higher *J*_SC_ and *V*_OC_ and improved FF. With the enhanced hole extraction and transport efficiency, the charge recombination at the perovskite/HTL interface is also well restricted, as confirmed by electrochemical impedance spectroscopy (EIS) (Fig. 4e). Notably, the pcz-SM-based device exhibits the largest charge recombination resistance (*R*_rec_) (480 Ω), indicating superior suppression of carrier recombination at the interfaces compared to pcz-SM-DM, pcz-DM and Spiro-OMeTAD-based devices. The pcz-SM-based PSCs show a weaker dependence of *V*_OC_ on light intensity (1.26 *kT*/*q*) (Fig. 4f), indicating that the improved *V*_OC_ is mainly due to suppressed Shockley–Read–Hall (SRH) recombination during device operation.^51^

**Fig. 4:**
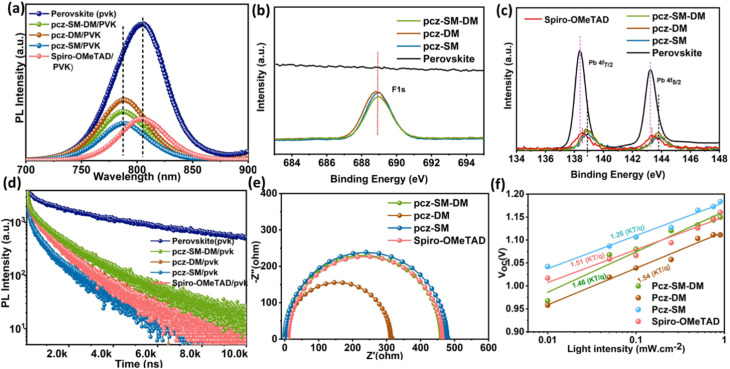
(a) Steady-state PL decay curves, (b) F1s XPS spectra and (c) Pb 4f XPS spectra of pristine perovskite, perovskite/pcz-SM-DM, perovskite/pcz-DM, perovskite/pcz-SM, and perovskite/Spiro-OMeTAD, (d) time-resolved PL decay results, (e) Nyquist plot, and (f) light intensity-dependent *V*_OC_ behavior of pcz-SM-DM, pcz-DM, and pcz-SM, and Spiro-OMeTAD.

The stability of PSCs with different HTMs (pcz-SM-DM, pcz-DM, pcz-SM and Spiro-OMeTAD) was monitored with unencapsulated devices under ambient (30–50% RH) conditions as shown in Fig. 5a. During the aging test, for pcz-SM-DM, pcz-DM and pcz-SM based PSCs, *J*_SC_, *V*_OC_, and FF gradually decrease, while the Spiro-OMeTAD based device showed a significant 20% drop in PCE within 450 h. After aging for 1000 h, the devices retained 73%, 62%, 81% and 65% of their initial PCE, respectively. The enhanced stability of pcz-SM is attributed to its homogeneous film nature, which acts as a passivating layer, protecting the perovskite layer from moisture-induced degradation, as discussed in the morphological analysis.

**Fig. 5:**
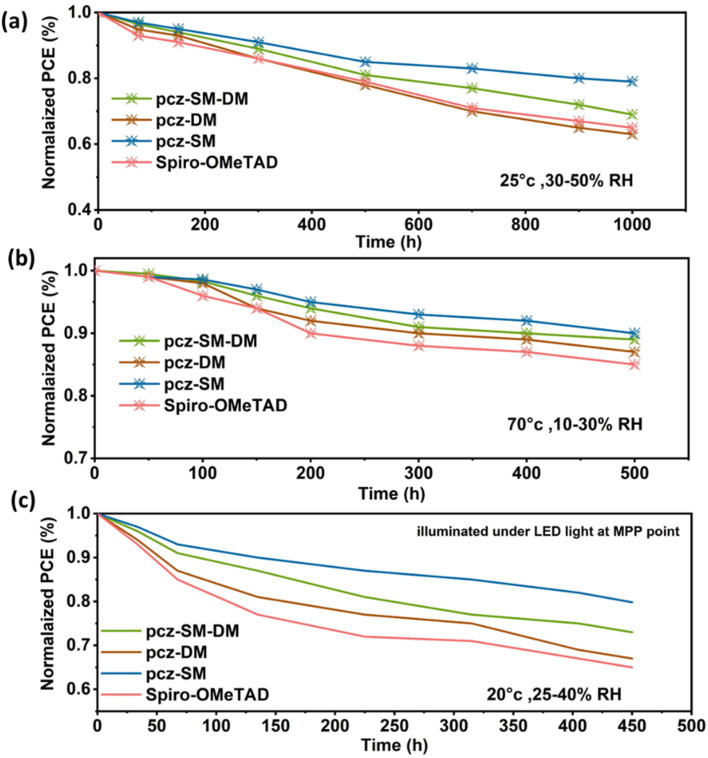
(a) Normalized humidity stability of PSCs, (b) thermal stability of PSCs, and (c) illumination stability of PSCs based on pcz-SM-DM, pcz-DM, and pcz-SM, and Spiro-OMeTAD devices.

Heating PSCs at a moderate temperature (70 °C) in a dry environment (10–30% RH) significantly slows down the device degradation. After 500 hours, all the devices retain at least 90% of their initial PCE (Fig. 5b). To assess the illumination stability of our devices, we exposed pcz-SM-DM, pcz-DM, and pcz-SM PSCs to ambient conditions of 25–40% RH while maintaining continuous illumination. Although all devices showed some degree of degradation over time (Fig. 5c), the pcz-SM-based device exhibited superior stability. Notably, this device retained an impressive 83% of its initial efficiency after the test, demonstrating its enhanced resilience under prolonged illumination. Therefore, pcz-SM is considered a promising low-cost alternative to Spiro-OMeTAD as an HTM in PSCs.

## Conclusion

By modifying phenoxazine-based HTMs with 1,3-di(trifluoromethyl)benzene and dimethyl-fluorene substitution, we developed three new defect-passivating HTMs for PSCs. HTM pcz-SM, with the most carbazole-dimethyl-fluorene end groups, prolonged the π conjugation length of the molecule and showed improved hole mobility and band alignment, leading to enhanced charge extraction and device performance (over 25% efficiency). The compact and pinhole-free morphology and defect-passivation effect of the pcz-SM film effectively suppress the charge recombination, leading to superior performance. This work demonstrates the effectiveness of peripheral group modification in improving charge carrier mobility and photovoltaic performance. The versatility of phenoxazine-based compounds offers opportunities for further optimization, making pcz-SM a promising HTM for large-scale, high-performance PSC applications.

## Author contributions

Murali Ravi: writing – original draft, methodology, investigation, data curation. Ziyang Xia: investigation, data curation. Divya Kumar: methodology, formal analysis. Cheng Chen: writing – review & editing, supervision, funding acquisition, formal analysis. Haoxin Wang: methodology, investigation. Yi Tian: writing – review & editing, formal analysis. Balamurali Ravichandran: formal analysis. Ming Cheng: writing – review & editing, supervision, methodology, funding acquisition.

## Conflicts of interest

There are no conflicts to declare.

## Supplementary Material

SC-OLF-D5SC04399A-s001

## Data Availability

The data supporting this article have been included as part of the SI. Supplementary information is available. See DOI: https://doi.org/10.1039/d5sc04399a.
