# Supplementary material for: Boosting planar perovskite solar cell performance via peripheral end-group engineering of phenoxazine-core hole transport materials
Source: Chem Sci. 2025 Sep 10;16(41):19205–14. doi: 10.1039/d5sc04399a (PMC12444427; doi:10.1039/d5sc04399a)
Supplement: SC-016-D5SC04399A-s001 [file SC-016-D5SC04399A-s001.pdf]

Supplementary Information (SI) for Chemical Science.
This journal is © The Royal Society of Chemistry 2025

Supporting Information

Boosting Planar Perovskite Solar Cell Performance via Peripheral End-Group Engineering of Phenoxazine-Core Hole Transport Materials

Synthesis method

Chemicals: All of the solvents and chemicals were used as received unless specially stated

Preparation of 10-(3,5-bis(trifluoromethyl)phenyl)-10H-phenoxazine (1)

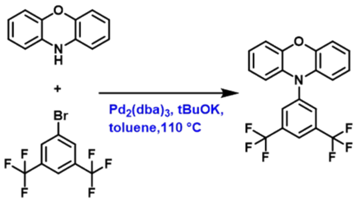

Under a N_2 atmosphere, a mixture of phenoxzaine (2mmol), and 1-bromo-3, 5-bis(trifluoromethyl)benzene (2mmol), palladium diacetate (0.1mmol), potassium tert butoxide (4 mmol), tri-tert-butylphosphine (0.2mmol), and solvent toluene (60mL) was placed in a two-necked 100 mL flask equipped with a reflux condenser After this, the mixture and the solvent was heated to 120 °C for 12 h. After that the reaction mixture was allowed to cool to room temperature and poured to water (40mL), followed by being extracted with ethyl acetate(EA) for several times. The combined organic layers were dried by anhydrous sodium sulfate (Na_2SO_4) and then concentrated. The residue was purified by column chromatography using a mixture of petroleum ether (PE)/EA (v/v= 2/1) as the eluent to give 10-(3,5-bis(trifluoromethyl)phenyl)-10H-phenoxazine (yield: 96 %) as the pale white flakes. ^{1}H NMR (400 MHz, $CDCl_3$) δ 8.04 – 8.01 (m, 1H), 7.85 – 7.83 (d, *J*

= 1.6 Hz, 2H), 6.91 – 6.89 (d, J = 2.2 Hz, 2H), 6.80 – 6.76 (dd, J = 8.5, 2.2 Hz, 2H), 5.74 – 5.68 (d, J = 8.5 Hz, 2H). ,

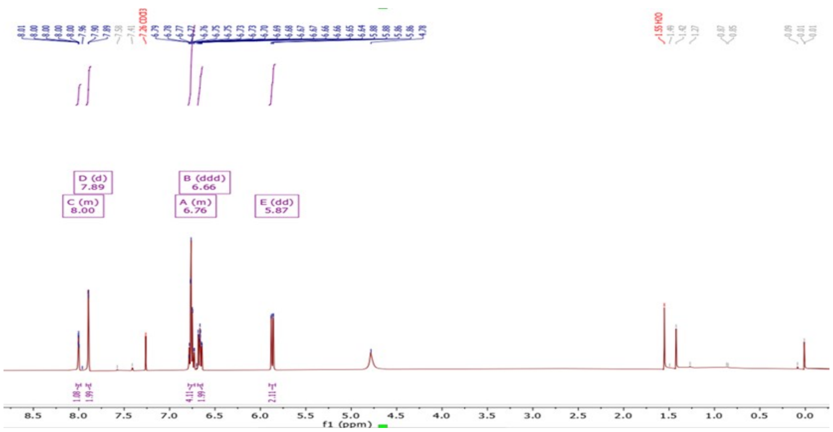

Figure. S1 ^{1}H NMR spectrum of 10-(3,5-bis(trifluoromethyl)phenyl)-10H-phenoxazine

Table S1 Synthesis cost of 10-(3,5-bis(trifluoromethyl)phenyl)-10H-phenoxazine **(1)**

**Reagent**	**Amount/g**	**Amount/mL**	**Price (RMB / g or RMB / mL)**	**Total price (RMB)**
phenoxzaine	0.9		56	50.4
1-bromo-3, 5-bis(trifluoromethyl)benzene	1.46		1.68	2.45
Tris(dibenzylideneacetone) di palladium	0.03		180	5.4
Tri-tert-butyl phosphonate		0.5	7.45	3.725
Potassium tert-butoxide	0.9		0.396	0.198
toluene		50	0.041	1.64
Pet ether	150		0.119	17.85

Silica gel	100.0		0.292	29.2
**Total cost**	110.84 RMB			
**Amount a-BTC**	1.78 g			
**COST for a-BTC**	64.7 RMB / g			
**Exchange rate**	1 $=6.974 RMB			

10-(3,5-bis(trifluoromethyl)phenyl)-3,7-dibromo-10H-phenoxazine (2)

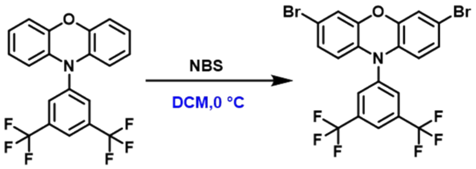

N-bromo-succinimide (10.5 mmol) was added in small portions to a solution of (**1**) (5 mmol) in DCM (50.0 mL) and the obtained mixture was stirred for 6 h at 0-5°C. The reaction mixture was then poured into water and the obtained solid was filtered, washed with water and dried under vacuum. The collected crude product was purified by column, elution through a short silica plug (petroleum ether / Ethyl acetate = 1:1.5 vol / vol) to obtain 2 yields: 85 % 1H NMR (400 MHz, CDCl3) δ 8.03 – 7.98 (m, 1H), 7.92 – 7.87 (d, J = 1.6 Hz, 2H), 6.79 – 6.72 (m, 4H), 6.69 – 6.64 (ddd, J = 7.9, 6.7, 2.3 Hz, 2H), 5.90 – 5.83 (dd, J = 7.9, 1.3 Hz, 2H).

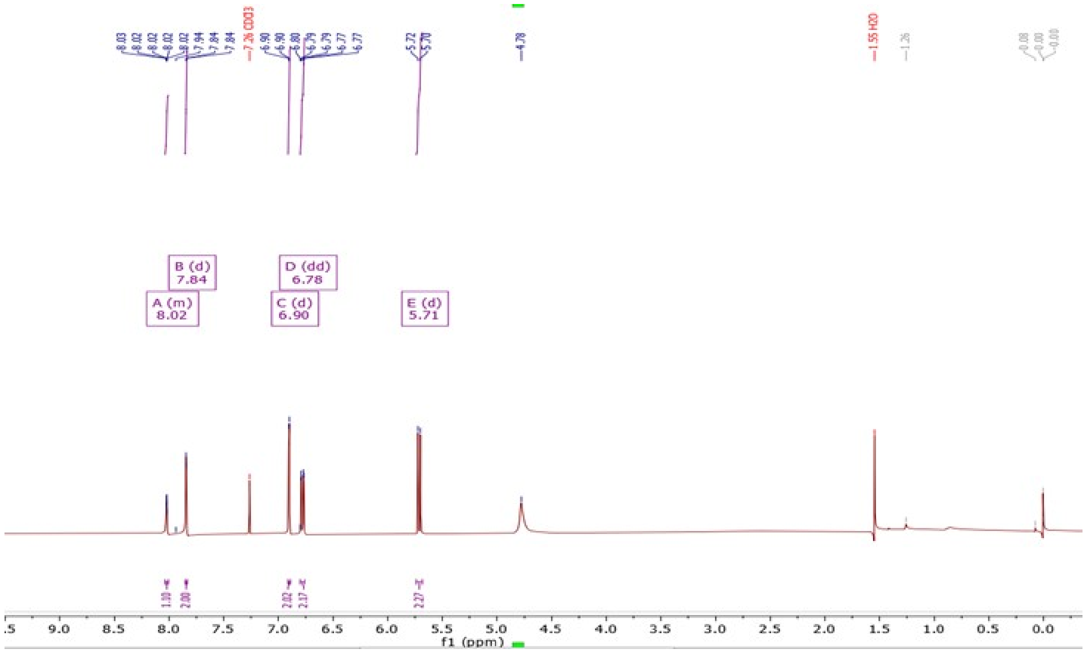

Figure. S2 ^{1}H NMR spectrum of 10-(3,5-bis(trifluoromethyl)phenyl)-3,7-dibromo-10H-phenoxazine

Table S2 Synthesis cost of 10-(3,5-bis(trifluoromethyl)phenyl)-3,7-dibromo-10H-phenoxazine (2)

Reagent	Amount / g	Amount / mL	Price (RMB/g or RMB/mL)	Total price (RMB)
1	2.7		64.7	174.6
THF		100.0	0.063	6.3
NBS	1.78		0.395	0.7
Silica gel	100.0		0.292	29.2
Petroleum ether		200.0	0.119	23.7
Dichloromethane		200.0	0.182	36.4
**Total cost**	270 RMB			
**Amount intermediate 2**	2.0 g			
**COST for intermediate 2**	135.1 RMB / g			

Exchange rate	1 $=6.974 RMB

N-(4-methoxyphenyl)-9,9-dimethyl-9H-fluoren-3-amine (3)

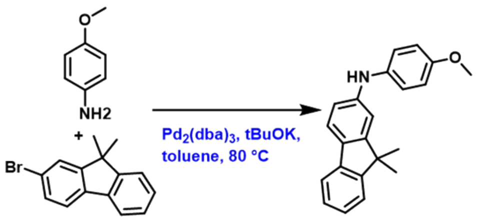

Under a N2 atmosphere, a mixture of 4-methoxyaniline (2 mmol), and 2-bromo-9,9-dimethyl-9H-fluorene (2 mmol), potassium tert butoxide (4 mmol), tri-tert-butylphosphine (0.2 mmol), and solvent toluene (60 mL) was placed in a two-necked 100 mL flask equipped with a reflux condenser and the solvent was heated to 80 °C for 4 h. The reaction mixture was allowed to cool to room temperature and poured to water (80 mL), followed by being extracted with Ethyl acetate for several times. The combined organic layers were dried by anhydrous sodium sulfate (Na_2SO_4) and then concentrated. The residue was purified by column chromatography using a mixture of petroleum ether (PE)/ Ethyl acetate (v/v= 2/1) as the eluent to give N-(4-methoxyphenyl)-9,9-dimethyl-9H-fluoren-3-amine (yield: 78 %) as the pale yellow powder. ^{1}H NMR (400 MHz, $CDCl_3$) δ 7.64 – 7.51 (m, 2H), 7.41 – 7.35 (dt, *J* = 7.2, 1.0 Hz, 1H), 7.32 – 7.27 (td, *J* = 7.4, 1.2 Hz, 1H), 7.25 – 7.18 (td, *J* = 7.4, 1.2 Hz, 1H), 7.15 – 7.09 (m, 2H), 7.01 – 6.97 (d, *J* = 2.2 Hz, 1H), 6.91 – 6.85 (dd, *J* = 8.6, 2.3 Hz, 3H), 5.65 – 5.58 (s, 1H), 3.86 – 3.78 (s, 3H), 1.49 – 1.41 (s, 6H).

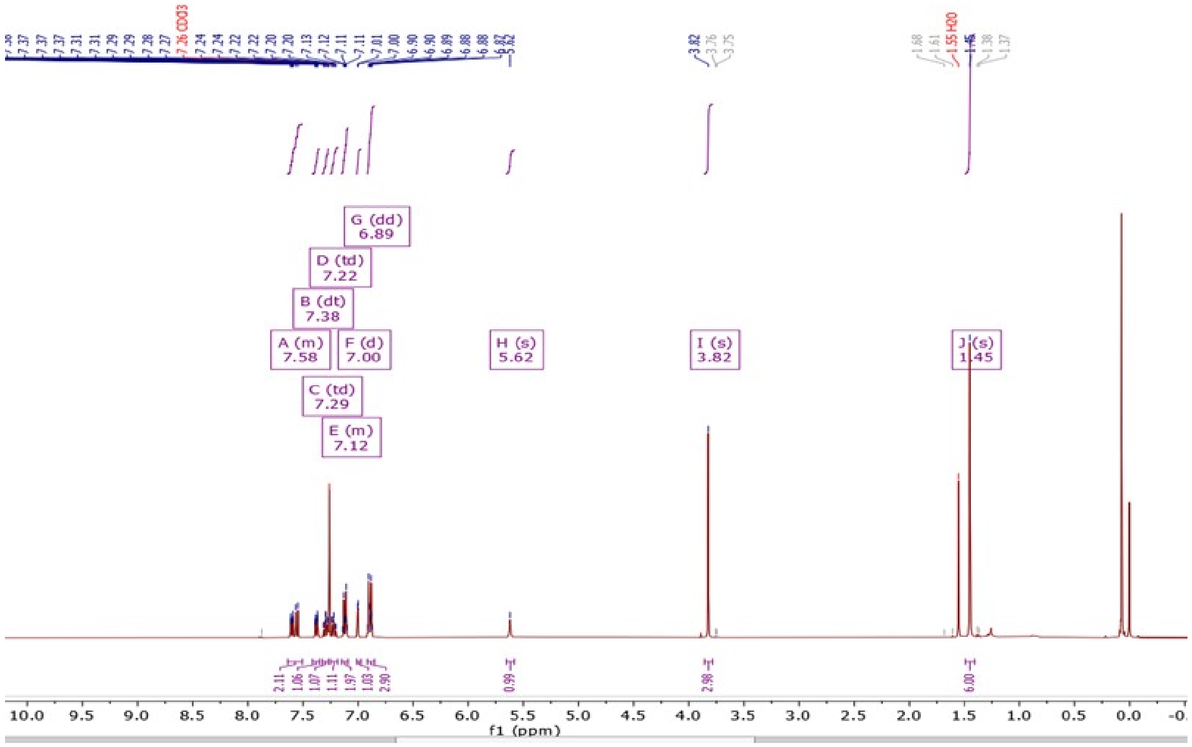

Figure. S3 ^{1}H NMR spectrum of N-(4-methoxyphenyl)-9,9-dimethyl-9H-fluoren-3-amine

Table S3 Synthesis cost of N-(4-methoxyphenyl)-9,9-dimethyl-9H-fluoren-3-amine (3)

Reagent	Amount / g	Amount / mL	Price (RMB / g or RMB / mL)	Total price (RMB)
4-methoxyaniline	1.23		5.32	6.5
2-bromo-9,9-dimethyl-9H-fluorene	2.73		3.08	8.40
Tris(dibenzylideneacetone)dipalladium	0.03		180	5.4
Tri-tert-butyl phosphonate		0.5	7.45	3.725
Potassium tert-butoxide	0.5		0.396	0.198
toluene		40	0.041	1.64

Pet ether	150		0.119	17.85
Silica gel	100.0		0.292	29.2
Ethyl acetate		200.0	0.063	12.6
**Total cost**	85.4 RMB			
**Amount intermediate 3**	3 g			
**COST for intermediate 3**	28.4 RMB / g			
**Exchange rate**	1 $=7.2 RMB			

N3,N3,N6,N6-tetrakis(4-methoxyphenyl)-9H-carbazole-3,6-diamine and N3,N6-bis(9,9-dimethyl-9H-fluoren-3-yl)-N3,N6-bis(4-methoxyphenyl)-9H-carbazole-3,6-diamine

A mixture of 3,6-dibromo-9H-carbazole (10 mmol), Di- tert -butyl decarbonate(15 mmol) in THF (20.0 mL) and add slowly 4-(Dimethylamino) pyridine(2 mmol) to the mixture was stirred at 70 °C for 2 h. After cooling down the reaction to the room temperature, the crude product was separated by the rotary evaporator to obtain (4-i) (yield: 97%) as a white solid no need column for these steps.

A mixture of (**4-i**) (11mmol), 4'4-dimethyl diphenylamine (22mmol), Tris(dibenzylideneacetone) dipalladium(0) (0.025mmol), tri-tert-butylphosphonate (0.05 mmol) and potassium tert-butoxide (10mmol) in toluene (80.0 mL) was stirred at 110°C for 16 h. After cooling down the reaction to room temperature, the mixture was diluted with dichloromethane and washed with water. The organic layer was collected and evaporated reduced pressure. The crude product was purified by column chromatography (petroleum ether / ethyl acetate = 1:0.5 vol / vol) to obtain **(4-ii)** (yield: 70%) as a pale-yellow solid.

A mixture of compound (**4-ii**) (5mmol), and potassium tert-butoxide (17mmol) in toluene (70.0 mL) stirred at 120℃ for 2h. After cooling down the reaction to the room temperature, the mixture was added 60.0 mL ethyl acetate in reaction solution, and then wash three times with 100.0 mL water. The organic layer was collected and evaporated reduced pressure. The crude product was purified by column chromatography (petroleum ether / ethyl acetate = 1:0.5 vol / vol) to obtain **(4)** (yield: 90.6%) as a yellow solid. **4-b** ^{1}H NMR (400 MHz, DMSO) δ 11.20 – 11.11 (s, 1H), 7.67 – 7.59 (s, 2H), 7.42 – 7.35 (d, *J* = 8.6 Hz, 2H), 7.08 – 7.01 (d, *J* = 8.6 Hz, 2H), 6.87 – 6.76 (t, *J* = 10.3 Hz, 16H), 3.70 – 3.65 (s, 12H). **4-a** ^{1}H NMR (400 MHz, DMSO) δ 11.34 – 11.30 (s, 1H), 7.88 – 7.83 (d, J = 2.2 Hz, 2H), 7.63 – 7.58 (d, J = 7.5 Hz, 2H), 7.56 – 7.53 (d, J = 8.4 Hz, 2H), 7.51 – 7.47 (d, J = 8.6 Hz, 2H), 7.43 – 7.40 (d, J = 7.4 Hz, 2H), 7.26 – 7.16 (m, 6H), 7.10 – 7.05 (m, 4H), 6.96 – 6.93 (d, J = 2.2 Hz, 2H), 6.90 – 6.86 (m, 4H), 6.73 – 6.69 (dd, J = 8.3, 2.2 Hz, 2H), 3.73 – 3.70 (s, 6H), 1.29 – 1.25 (s, 12H).

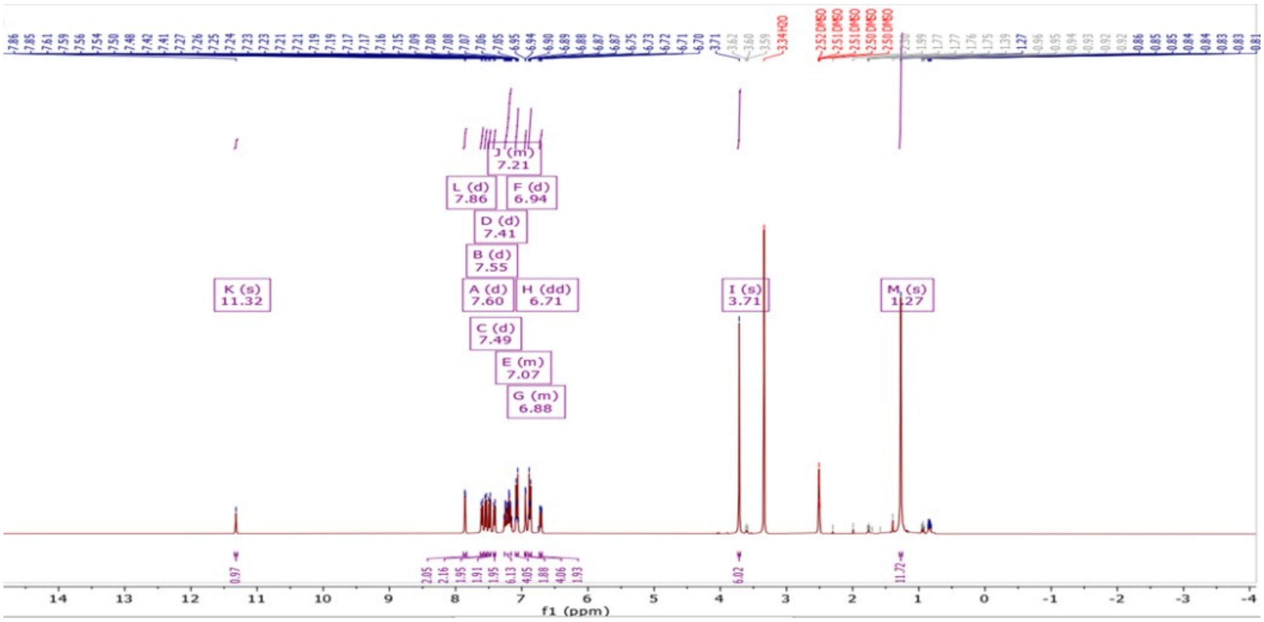

Figure. S4 ^{1}H NMR spectrum of N3,N3,N6,N6-tetrakis(4-methoxyphenyl)-9H-carbazole-3,6-diamine (**4-a**)

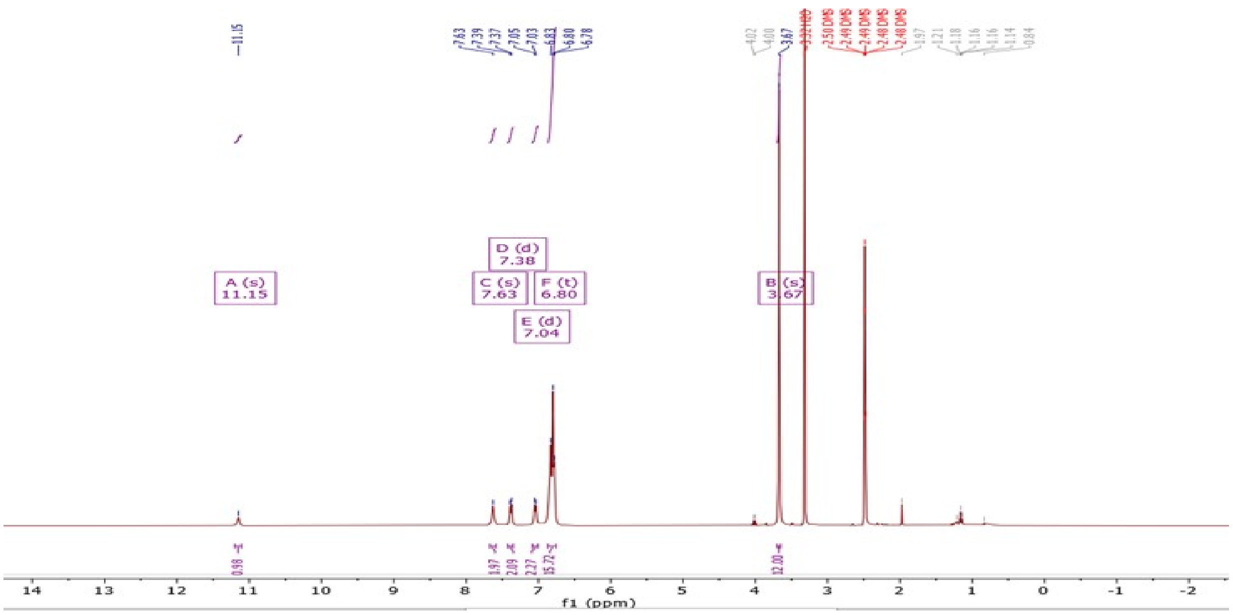

Figure. S5 ^{1}H NMR spectrum of N3,N6-bis(9,9-dimethyl-9H-fluoren-3-yl)-N3,N6-bis(4-methoxyphenyl)-9H-carbazole-3,6-diamine (**4-b**)

9,9'-(10-(3,5-bis(trifluoromethyl)phenyl)-10H-phenoxazine-3,7-diyl)bis(N3,N3,N6,N6-tetrakis(4-methoxyphenyl)-9H-carbazole-3,6-diamine) (pcz-DM)

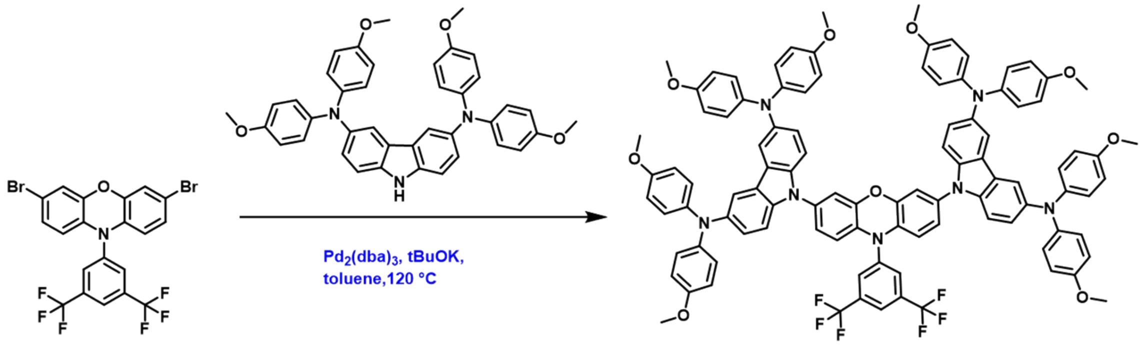

A mixture of (**4-b**) (1mmol), **(2)** (2.2mmol), Tris(dibenzylideneacetone) dipalladium(0) (0.025mmol), tri-tert-butylphosphonate (0.05 mmol) and potassium tert-butoxide (10mmol) in toluene (80.0 mL) was stirred at 110°C for 16 h. After cooling down the reaction to room temperature, the mixture was diluted with ethyl acetate and washed with water. The organic layer was collected and evaporated reduced pressure. The crude product was purified by column chromatography (petroleum ether / ethyl acetate = 1:0.5 vol / vol) to obtain pcz-DM (yield: 74%)

as a pale-yellow solid. 1H NMR (400 MHz, CDCl3) δ 8.11 – 8.04 (d, J = 13.0 Hz, 2H), 7.68 – 7.61 (dd, J = 15.8, 2.2 Hz, 4H), 7.30 – 7.23 (d, J = 8.8 Hz, 6H), 7.16 – 7.11 (dd, J = 8.8, 2.2 Hz, 3H), 7.08 – 6.93 (m, 18H), 6.92 – 6.72 (m, 18H), 3.85 – 3.67 (d, J = 1.8 Hz, 24H).^{13}C NMR (101 MHz, DMSO) δ 154.86 – 154.67, 144.05 – 144.00, 143.08 – 143.01, 142.52 – 142.32, 142.26 – 142.22, 141.71 – 141.65, 137.71 – 137.59, 132.93 – 132.82,131.49 – 131.39, 124.64 – 124.15, 123.73 – 123.59, 116.98 – 116.89,115.51 – 114.91 11.15 – 111.09 (d, *J* = 9.2 Hz) , 55.80 – 55.23. HR-MS: calculated: $C_{100}H_{77}F_6N_7O_9$ 1,633.5681, found: 1,633.5761

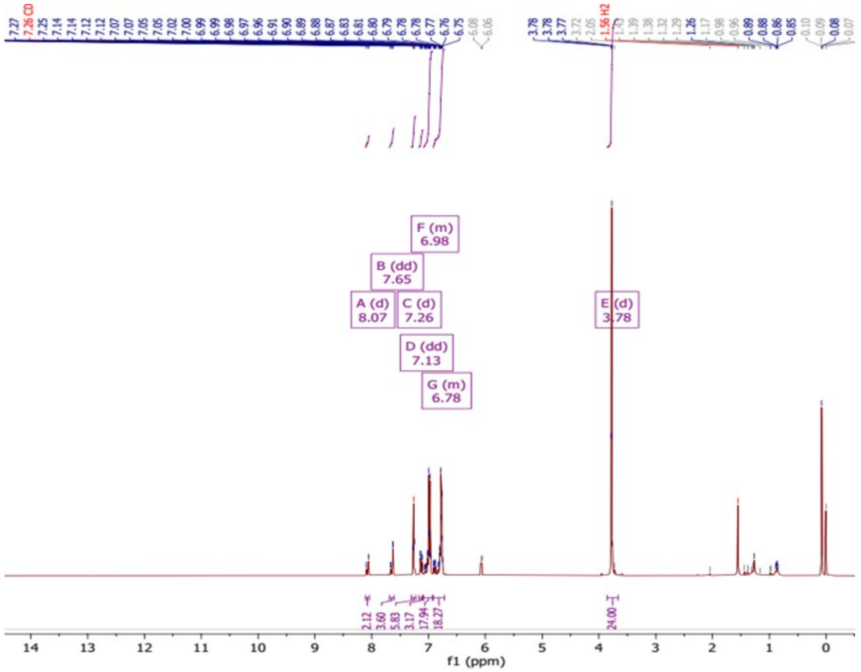

Figure. S6 ^{1}H NMR spectrum of pcz-DM

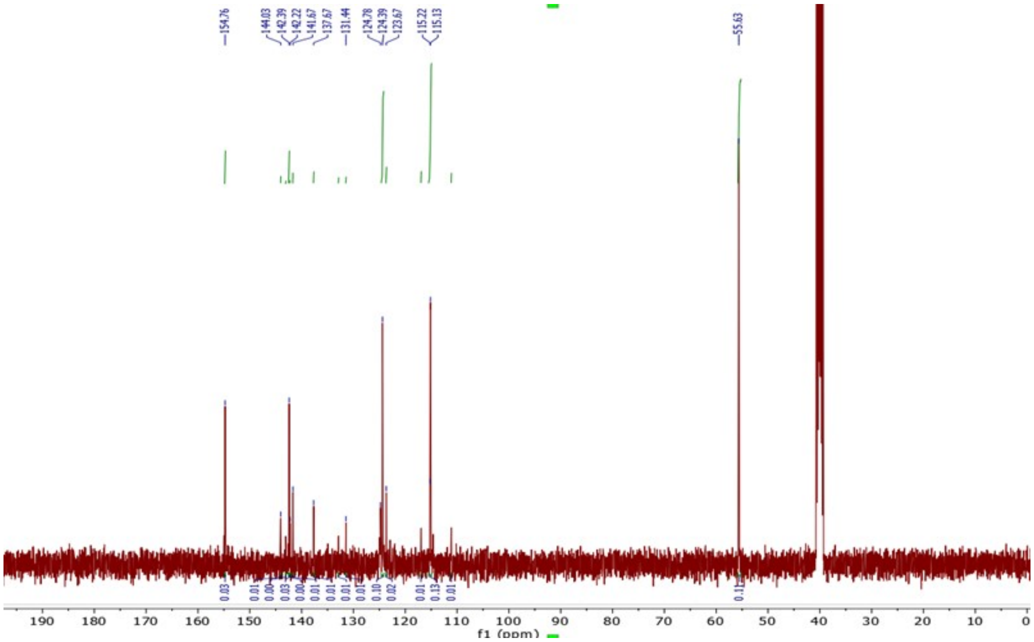

Figure. S7. ^{13}C NMR spectrum of pcz-DM

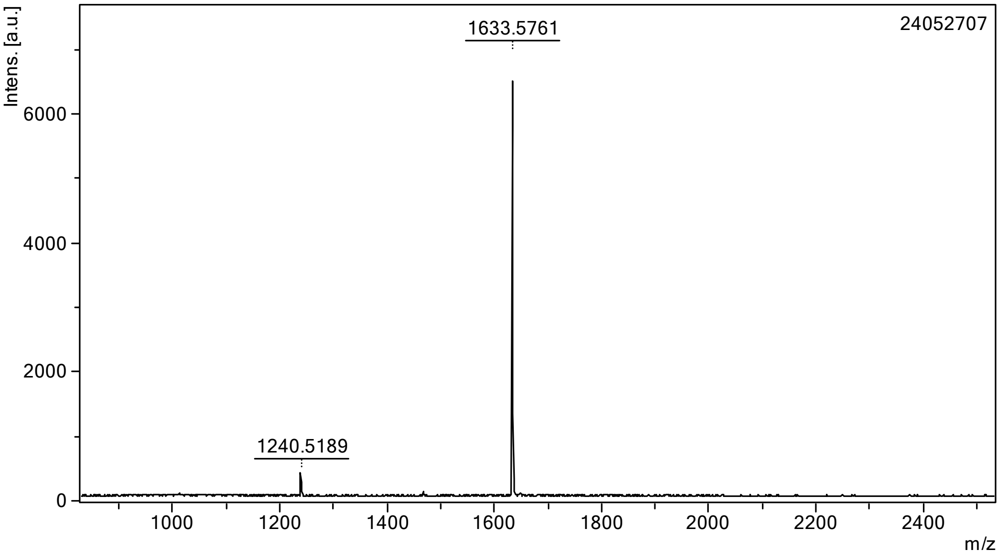

Figure. S8 Mass spectrum of pcz-DM

Table S4 Synthesis cost of pcz-DM

**Reagent**	**Amount/g**	**Amount/mL**	**Price (RMB / g or RMB / mL)**	**Total price (RMB)**

intermediate 4-b	1.242		77.3	92.76
intermediate 2	0.5		135.1	67.53
Tris(dibenzylideneacetone)dipalladium	0.03		180	5.4
Tri-tert-butyl phosphonate		0.5	7.45	3.725
Potassium tert-butoxide	0.5		0.396	0.198
toluene		40	0.041	1.64
Pet ether	150		0.119	17.85
Silica gel	100.0		0.292	29.2
Ethyl acetate		200.0	0.063	12.6
**Total cost**	230.8 RMB			
**Amount pcz-DM**	1.1g			
**COST for pcz-DM**	209.8 RMB / g			
**Exchange rate**	1 $=7.2 RMB			

9-(10-(3,5-bis(trifluoromethyl)phenyl)-7-bromo-10H-phenoxazin-3-yl)-N3,N3,N6,N6-tetrakis(4-methoxyphenyl)-9H-carbazole-3,6-diamine (5)

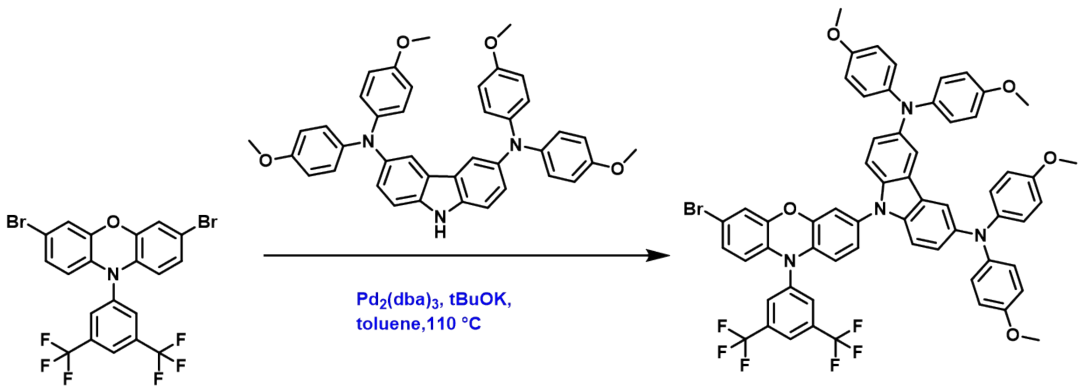

A mixture of **(3a)** (1mmol), **(2a)** (1mmol), Tris(dibenzylideneacetone) dipalladium(0) (0.025mmol), tri-tert-butylphosphonate (0.05 mmol) and potassium tert-butoxide (10mmol) in toluene (80.0 mL) was stirred at 110°C for 12 h. After cooling down the reaction to room temperature, the mixture was diluted with dichloromethane and washed with water. The organic layer was collected and evaporated reduced pressure. The crude product was purified by column chromatography (petroleum ether / ethyl acetate = 1:0.5 vol / vol) to obtain (4) (yield: 58%) as a pale-yellow solid. 1H NMR (400 MHz, DMSO) δ 8.06 – 8.01 (s, 1H), 7.97 – 7.91 (d, J = 8.6 Hz, 2H), 7.63 – 7.58 (d, J = 2.1 Hz, 2H), 7.24 – 7.19 (m, 2H), 7.14 – 7.07 (dd, J = 8.8, 2.2 Hz, 2H), 7.00 – 6.90 (m, 10H), 6.86 – 6.82 (dd, J = 8.7, 2.3 Hz, 1H), 6.81 – 6.72 (m, 10H), 6.03 – 5.96 (d, J = 8.5 Hz, 1H), 3.78 – 3.73 (s, 12H).

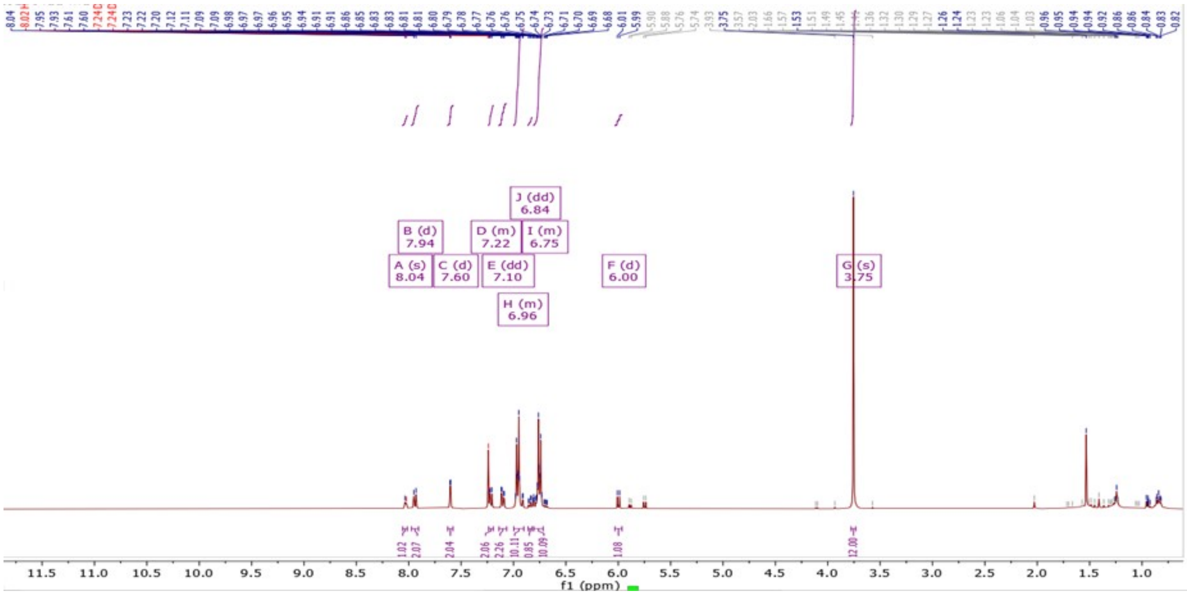

Figure. S9 ^{1}H NMR spectrum of 9-(10-(3,5-bis(trifluoromethyl)phenyl)-7-bromo-10H-phenoxazin-3-yl)-N3,N3,N6,N6-tetrakis(4-methoxyphenyl)-9H-carbazole-3,6-diamine (5)

Table S5 Synthesis cost of (5)

Reagent	Amount / g	Amount / mL	Price (RMB/g or RMB/mL)	Total price (RMB)
intermediate 4-b	1.242		77.3	92.76
intermediate 2	1.1		135.7	149.1
Tris(dibenzylideneacetone)dipalladium	0.03		180	5.4
Tri-tert-butyl phosphonate		0.5	7.45	3.725
Potassium tert-butoxide	0.5		0.396	0.198
toluene		40	0.041	1.64
Pet ether	150		0.119	17.85
Silica gel	100.0		0.292	29.2
Ethyl acetate		200.0	0.063	12.6
**Total cost**	312.20 RMB			
**Amount of intermediate5**	1.2g			
**COST for intermediate 5**	260.2 RMB / g			
**Exchange rate**	1 $=7.2 RMB			

9-(7-(3,6-bis((9,9-dimethyl-9H-fluoren-3-yl)(4-methoxyphenyl)amino)-9H-carbazol-9-yl)-10-(3,5-bis(trifluoromethyl)phenyl)-10H-phenoxazin-3-yl)-N3,N3,N6,N6-tetrakis(4-methoxyphenyl)-9H-carbazole-3,6-diamine (pcz-SM-DM)

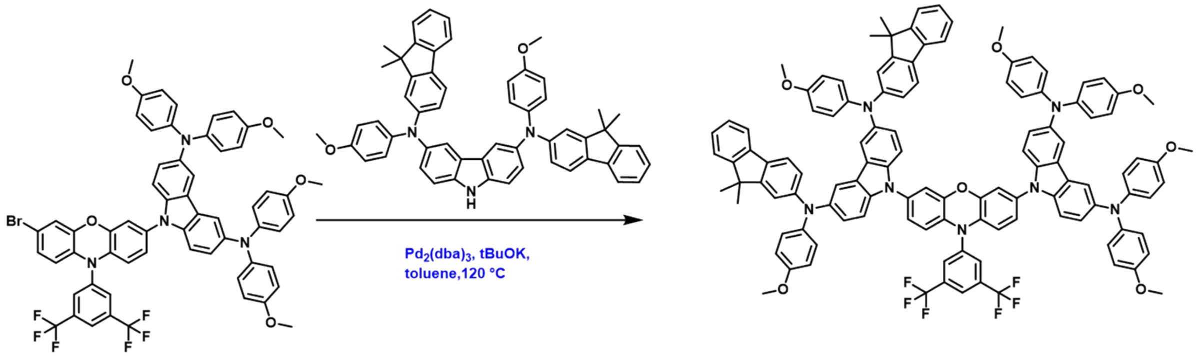

A mixture of **(3b)** (11mmol), **(4)** (1mmol), Tris(dibenzylideneacetone) dipalladium(0) (0.025mmol), tri-tert-butylphosphonate (0.05 mmol) and potassium tert-butoxide (10mmol) in toluene (80.0 mL) was stirred at 110°C for 12 h. After cooling down the reaction to room temperature, the mixture was diluted with dichloromethane and washed with water. The organic layer was collected and evaporated reduced pressure. The crude product was purified by column chromatography (petroleum ether / ethyl acetate = 1:0.5 vol / vol) to obtain pcz-SM-DM (yield: 66.9%) as a pale-yellow solid ^{1}H NMR (400 MHz, DMSO) δ 8.59 – 8.48 (s, 2H), 7.88 – 7.82 (m, 2H), 7.71 – 7.65 (s, 2H), 7.61 – 7.52 (dd, J = 21.7, 7.9 Hz, 5H), 7.50 – 7.35 (m, 4H), 7.35 – 7.28 (d, J = 8.8 Hz, 2H), 7.27 – 7.11 (m, 9H), 7.10 – 6.98 (m, 8H), 6.98 – 6.89 (dd, J = 14.8, 2.2 Hz, 4H), 6.90 – 6.73 (m, 17H), 6.72 – 6.65 (dd, J = 8.4, 2.1 Hz, 2H), 6.15 – 6.05 (m, 2H), 3.72 – 3.49 (m, 18H), 1.28 – 1.23 (d, J = 2.3 Hz, 12H).^{13}C NMR (101 MHz, DMSO) δ 156.28 – 155.44, 155.11 – 153.34 – 152.84, 149.22 – 148.74, 142.58 – 142.12, 141.86 – 141.13, 139.54 – 138.90, 138.31 – 137.69, 133.53 – 133, 126.83 – 126.10, 124.84 – 123.9, 123.07 – 122.62, 121.31 – 120.49, 119.62 – 118.96, 115.64 – 114.70, 114.47 – 112.85, 111.44 – 110.81, 56.09 – 55.08, 47.55 – 46.20, 27.77 – 27.21. HR-MS: calculated: C_{116} $H_{89}F_6N_7O_7$ .1,805.6722, found: 1,805.6696

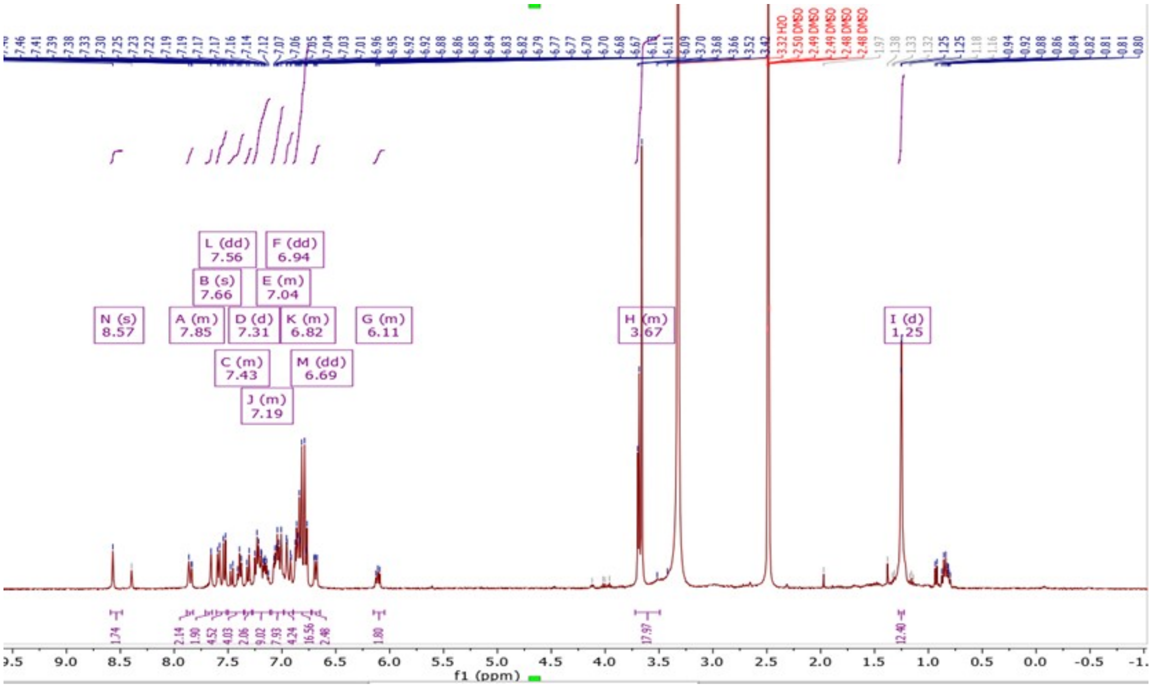

Figure. S10 ^{1}H NMR spectrum of pcz-SM-DM

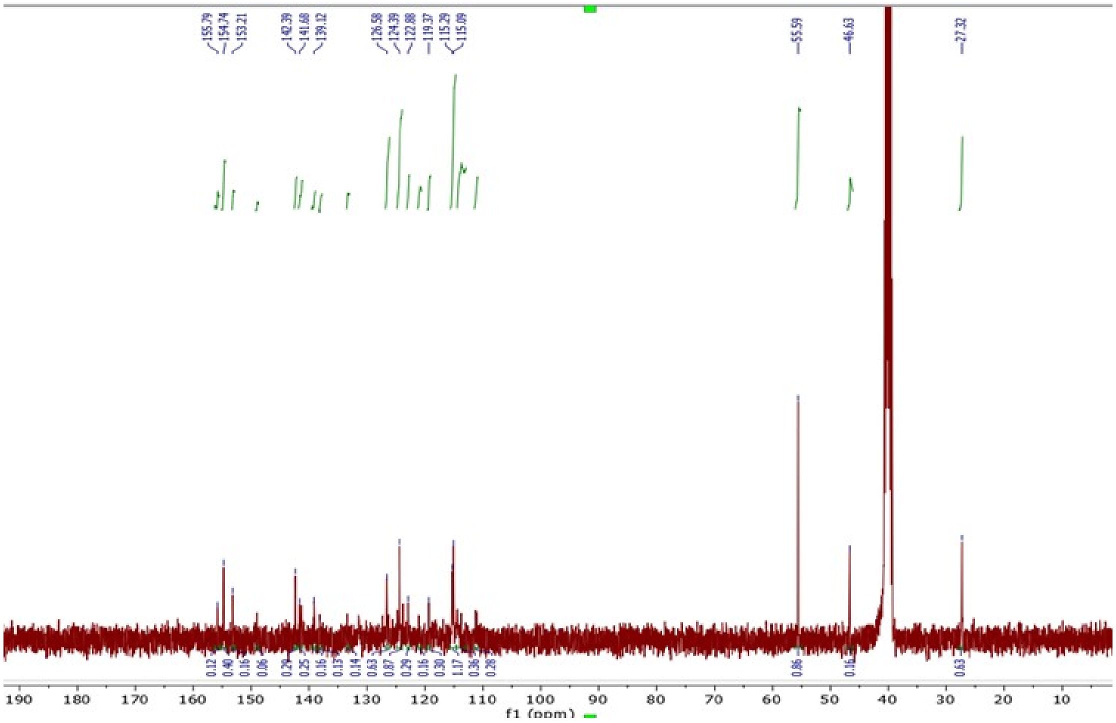

Figure. S11. ^{13}C NMR spectrum of pcz-SM-DM

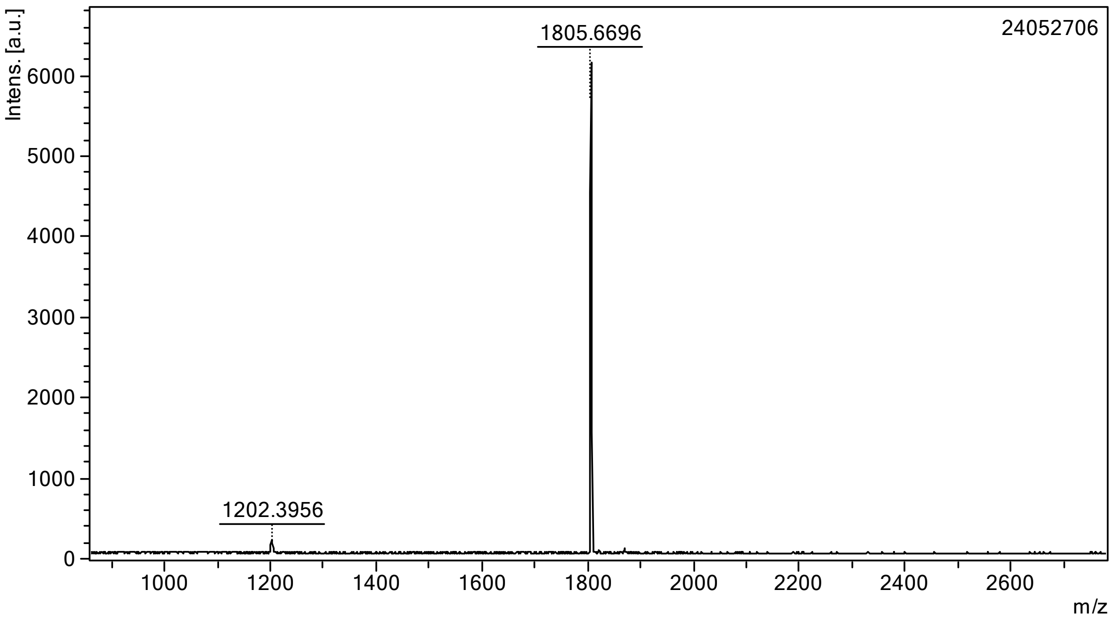

Figure. S12 Mass spectrum of pcz-SM-DM

Table S6 Synthesis cost of pcz-SM-DM

Reagent	Amount/g	Amount/mL	Price (RMB / g or RMB / mL)	Total price (RMB)
intermediate 4-a	0.4		92	36.8
intermediate 5	0.54		260.2	140.5
Tris(dibenzylideneacetone)dipalladium	0.03		180	5.4
Tri-tert-butyl phosphonate		0.5	7.45	3.725
Potassium tert-butoxide	0.5		0.396	0.198
toluene		40	0.041	1.64
Pet ether	150		0.119	17.85
Silica gel	100.0		0.292	29.2

Ethyl acetate		200.0	0.063	12.6
**Total cost**	247.0 RMB			
**Amount pcz-SM-DM**	0.5g			
**COST for pcz-SM-DM**	495.0RMB / g			
**Exchange rate**	1 $=7.2 RMB			

9,9'-(10-(3,5-bis(trifluoromethyl)phenyl)-10H-phenoxazine-3,7-diyl)bis(N3,N6-bis(9,9-dimethyl-9H-fluoren-3-yl)-N3,N6-bis(4-methoxyphenyl)-9H-carbazole-3,6-diamine)

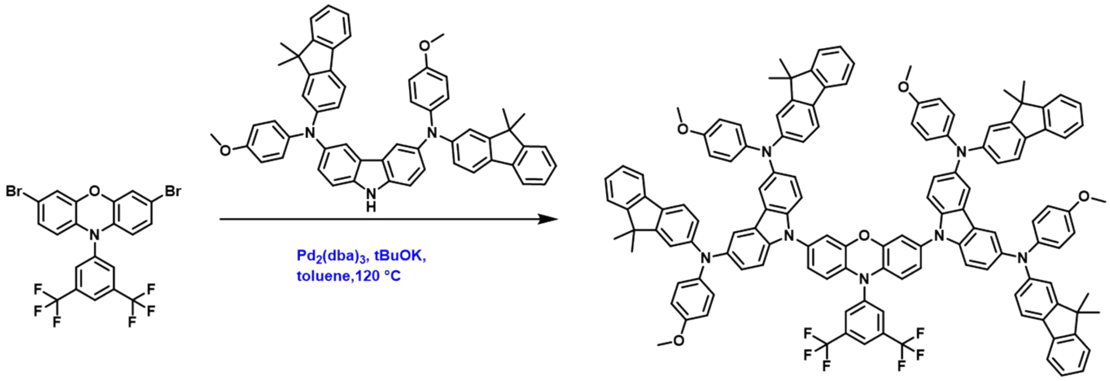

A mixture of **(2)** (1mmol), **(4a)** (2mmol), Tris(dibenzylideneacetone) dipalladium(0) (0.025mmol), tri-tert-butylphosphonate (0.05 mmol) and potassium tert-butoxide (10mmol) in toluene (80.0 mL) was stirred at 120°C for 16 h. After cooling down the reaction to room temperature, the mixture was diluted with ethyl acetate and washed with water. The organic layer was collected and evaporated reduced pressure. The crude product was purified by column chromatography (petroleum ether / ethyl acetate = 1:0.5 vol / vol) to obtain pcz-SM (yield: 78.1%) as a pale-yellow solid ^{1}H NMR (400 MHz, CDCl3) δ 8.11 – 8.07 (d, J = 11.8 Hz, 2H), 7.76 – 7.71 (d, J = 2.1 Hz, 4H), 7.60 – 7.55 (d, J = 7.4 Hz, 4H), 7.50 – 7.46 (d, J = 8.3 Hz, 4H), 7.36 – 7.30 (dd, J = 8.1, 2.8 Hz, 8H), 7.28 – 7.21 (m, 13H), 7.13 – 7.05 (m, 14H), 6.98 – 6.94 (dd, J = 8.5, 2.3

Hz, 2H), 6.92 – 6.88 (dd, J = 8.3, 2.2 Hz, 4H), 6.84 – 6.79 (m, 8H), 6.14 – 6.08 (d, J = 8.5 Hz, 2H), 3.83 – 3.75 (s, 12H), 1.38 – 1.32 (s, 24H).^{13}C NMR (101 MHz, DMSO) δ 155.97 – 155.47, 153.23 – 152.93, 149.13 – 148.73, 144.17 – 143.81, 141.49 – 140.71, 139.33 – 138.83, 138.37 – 137.79, 131.1 – 130.73, 127.63 – 125.59, 124 – 123.27, 123.13 – 122.31, 121.47 – 120.65, 119.76 – 118.95, 115.61 – 114.71, 114.71 – 114.17, 111.63 – 110.51, 56.30 – 54.57, 47.49 – 45.76, 27.84 – 26.86. HR-MS: calculated: $C_{132}H_{101}F_6N_7O_5$ 1,977.7763, found: 1,977.7715.

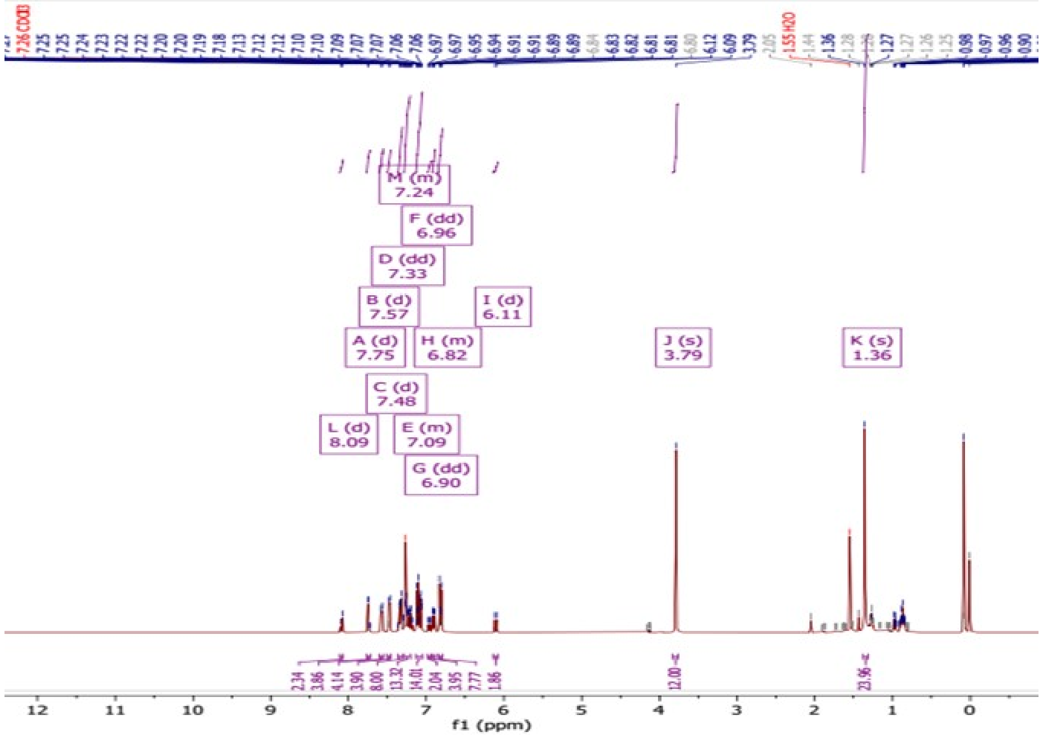

Figure. S13 ^{1}H NMR spectrum of pcz-SM

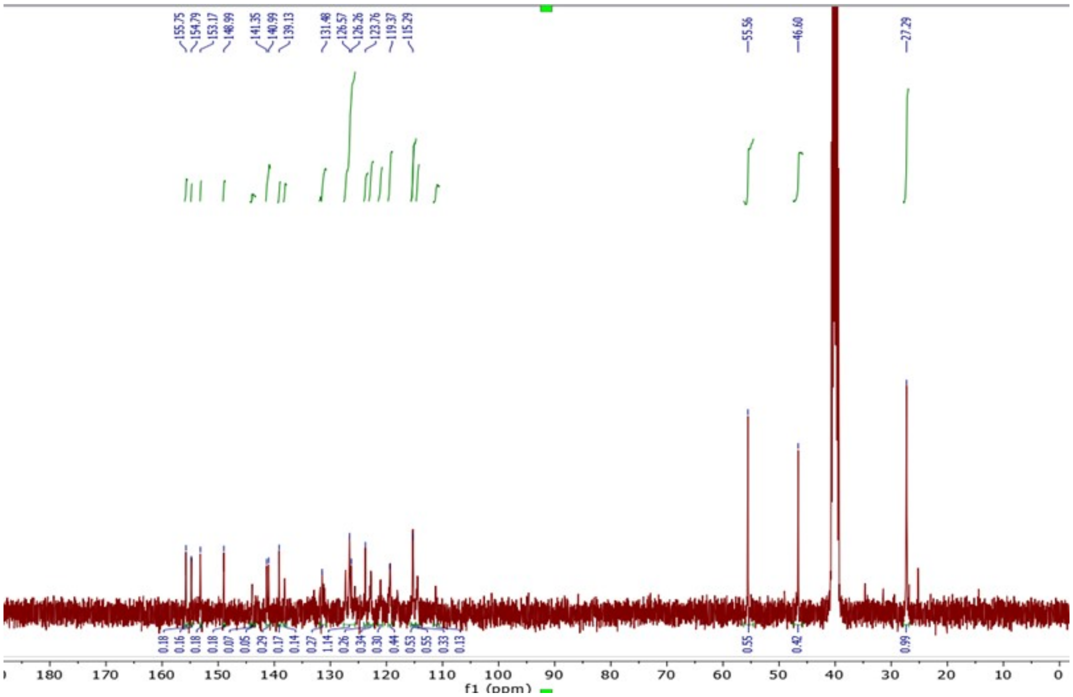

Figure. S14. ^{13}C NMR spectrum of pcz-SM

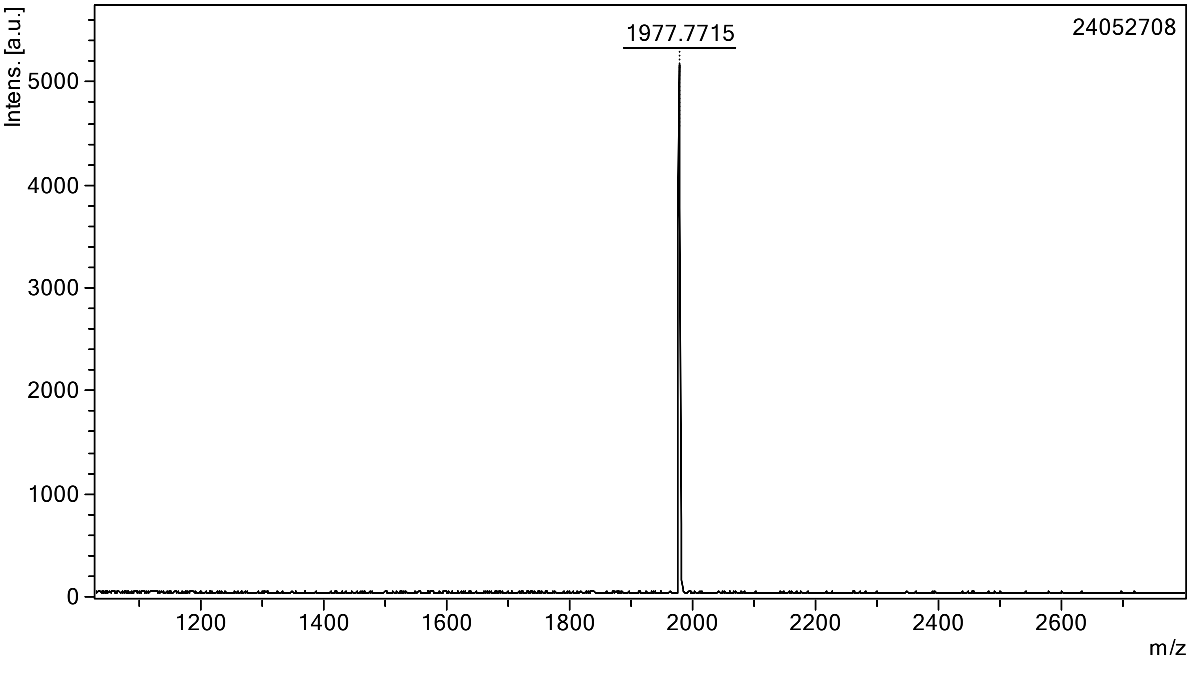

Figure. S15 Mass spectrum of pcz-SM

Table S7 Synthesis cost of pcz-SM

Reagent	Amount / g	Amount / mL	Price (RMB / g or RMB / mL)	Total price (RMB)
intermediate 4-a	0.8		92	73.6
intermediate 5	0.27		135.6	36.61
Tris(dibenzylideneacetone)dipalladium	0.03		180	5.4
Tri-tert-butyl phosphonate		0.5	7.45	3.725
Potassium tert-butoxide	0.5		0.396	0.198
toluene		40	0.041	1.64
Pet ether	150		0.119	17.85
Silica gel	100.0		0.292	29.2
Ethyl acetate		200.0	0.063	12.6
**Total cost**	180.7 RMB			
**Amount pcz-SM**	0.7g			
**COST for pcz-SM**	258.6RMB / g			
**Exchange rate**	1 $=7.2 RMB			

EXPERIMENTAL PROCEDURES

In the simulation for this publication optimization and single point energy calculations are performed using the B3LYP and the 6-31G* basis set for all atoms, without any symmetry constraints. All reported calculations were carried out by means of Gaussian 09 Measurement

The absorption spectra were recorded on an Agilent 8453 spectrophotometer using a 1 cm cuvette. Cyclic voltammetry (CV) was performed in dichloromethane with 0.1 M $TBAPF_6$ as the supporting electrolyte, a $Ag^+/AgNO_3$ electrode as the reference electrode, a carbon-glass electrode as the working electrode, a Pt electrode as the counter electrode and ferrocene/ferrocenium (Fc/Fc^+) as an internal reference with a CH Instruments electrochemical workstation (model 660 A). The SEM images were taken on a JEOL JSM-S4800. Light source for the photocurrent-voltage (*J–V*) measureme17nt is an AM 1.5G solar simulator. The incident light intensity was 100 mW·cm^{-2} calibrated with a standard Si solar cell. The tested solar cells were masked to a working area of 0.126 cm^2. The photocurrent-voltage (*J–V*) curves were obtained by the linear sweep voltammetry (LSV) method using a Keithley 2400 source-measure unit. The measurement of the incident-photon-to-current conversion efficiency (IPCE) was performed with CEL-QPCE3000 photoelectric chemical quantum efficiency testing and analysis system.

Hole mobility was measured by using the space-charge-limited current (SCLC) method with the device structure of ITO/PEDOT:PSS/HTM/Au. Space-charge-limited current can be described by equation below:

$$J = \frac{9}{8}\mu\varepsilon_0\varepsilon_r\frac{V^2}{D^3}$$

where J is the current density, μ is the hole mobility, ε_o is the vacuum permittivity (8.85×10^{-12} F / m), ε_r is the dielectric constant of the material (normally taken to approach 3 for organic semiconductors), V is the applied bias, and D is the film thickness.

The electrical conductivities of the HTM films were determined by using two-probe electrical conductivity measurements. The electrical conductivity (σ) was calculated by using the following equation:

$$\sigma = \frac{W}{R\,L\,D}$$

where L is the channel length 10 mm, W is the channel width 2 mm, D is the film thickness of the TiO_2 and HTM, and R is the film resistance calculated from the gradients of the curves.

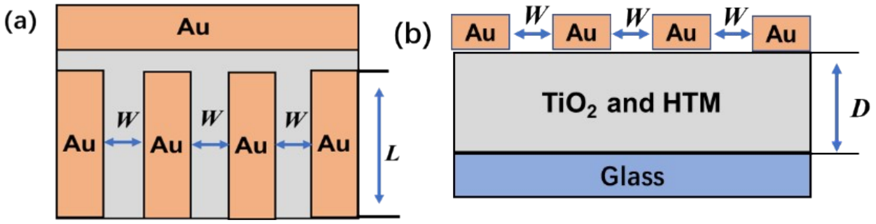

Schematic illustrations of the conductivity device: (a) top-sectional view; (b) cross-sectional view

XPS measurement details. obtained using a Thermo Scientific EscaLab 250Xi spectrometer. The analysis was conducted with a monochromatic Al Kα X-ray source (1486.6 eV, 150 W) operating in hybrid lens mode (160 µm spot size), utilising both survey scans (100 eV pass energy, 1000 meV step size) and high-resolution regional scans (20 eV pass energy, 50 meV step size). Charge compensation was maintained using a flood gun (1.7 A current, 3.3 V balance, 1.3 V bias), with all measurements performed under ultra-high vacuum conditions. Energy calibration was consistently referenced to adventitious carbon (C 1s at 284.8 eV), and each scan was acquired for 121 seconds to ensure optimal signal-to-noise ratio while minimising sample damage.

Ultraviolet photoelectron spectroscopy (UPS) is conducted to investigate the energy level, combined with the secondary cutoff edge (E_{cutoff}),

$$\mathrm{WF} = E_{\mathrm{cut\ off}} - hv$$

$$\mathrm{VBM} = \mathrm{WF} - E_{\mathrm{onset}}$$

WF can be obtained according to WF = 21.22 − E_{cutoff}.

Perovskite Solar Cell Fabrication

The perovskite and hole-transport material solutions were prepared inside an Argon glovebox. Conducting SnO_2, glass substrates (Pilkington, TEC15) were cut (25 mm x 15 mm) and patterned by chemical etching using zinc powder and hydrochloric acid. The substrates were washed by sonication subsequentially in 2% RBS™50 solution (Fluka) for 30 minutes, deionized water, acetone and ethanol for 15 minutes each. A 20~30 nm SnO_2 layer was then deposited on the cleaned FTO glass. The perovskite solution was kept at room temperature at all times to dissolve. 30 µl of the perovskite solution was spread onto the SnO_2. The substrate was spin-coated at 1000 rpm for 10 seconds and 4000 rpm for 30 seconds with a ramp speed of 2000 rpm/s. During the second spin-coating step, an anti-solvent was injected onto the film after 15 seconds using 100 µl of chlorobenzene (anhydrous, Aldrich). The perovskite films were then annealed at 120°C for 60 minutes on a hotplate after cool down. Subsequently, the HTM layer was then spin-coated on top of the perovskite film. The HTL (~150 nm) was deposited by spin coating at 4000 rpm for 30 s with 1 mL of chlorobenzene solution at room temperature (25°C) [4]. (Spiro-OMeTAD: 70 mg Spiro-OMeTAD, 30 mM LiTFSI , 250 mM TBP; pcz-SM-DM: 55 mg pcz-SM-DM, 250 mM TBP, 25 mM LiTFSI,; pcz-DM: 55 mg pcz-DM, 25 mM LiTFSI, 250 mM TBP, pcz-SM: 55 mg pcz-SM, 25 mM LiTFSI, 250 mM TBP). Finally, a layer of 100 nm Au was deposited sequentially under high vacuum ($<4 \times 10^{-4}$ Pa) by thermal evaporation through a shadow mask to form an active area of ~20 mm^2.

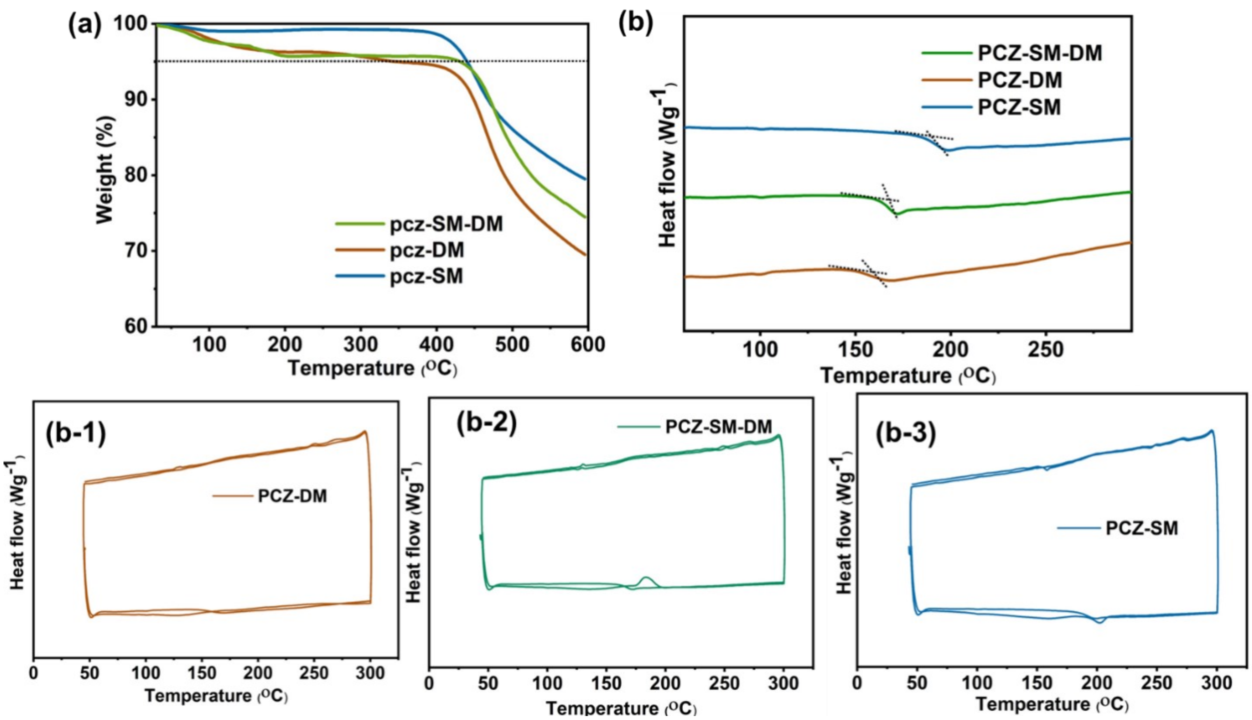

Figure.S16 d) TGA and (b) 2nd Heating cycle of DSC plot ;(b-1-b-3) Two heating and cooling DSC cycle f of pcz-SM-DM, pcz-DM, and pcz-SM HTMs, the heating rate is 10 ^{o}C min^{-1}.

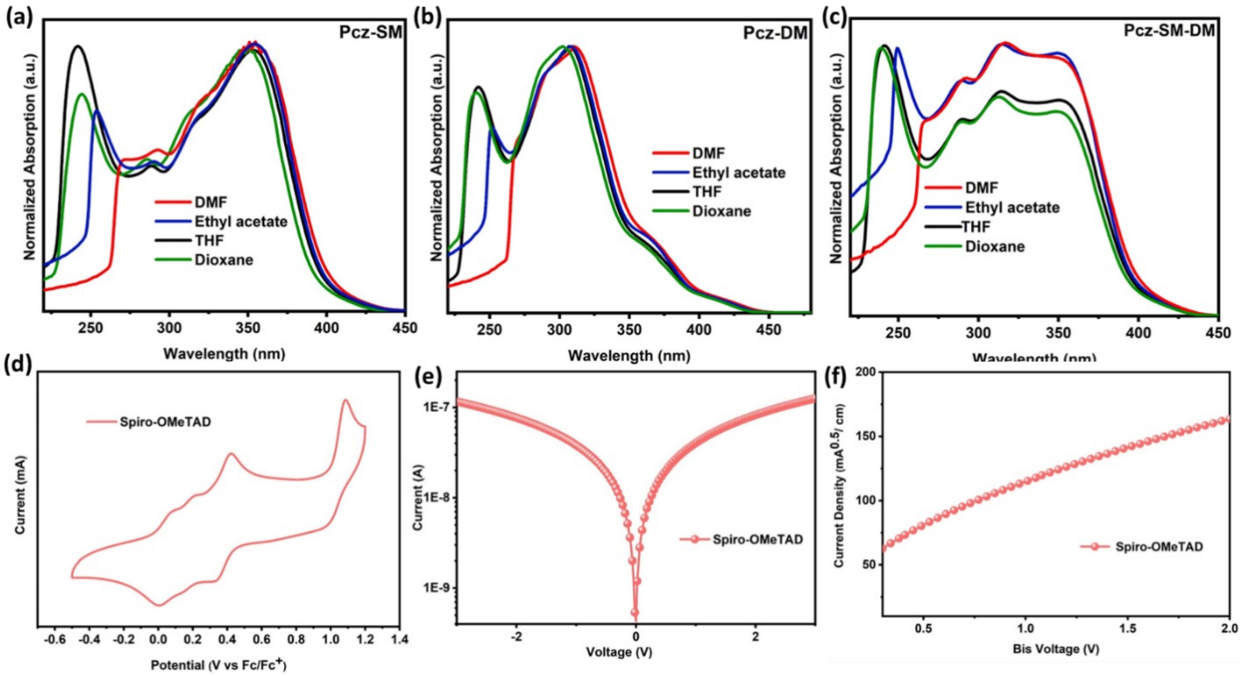

Figure.S17 (a-c) UV absorption spectrum of HTMs in different polar solvent (d) CV curves analyses in DCM (1.0×10^{-3} M) (e) Hole mobility and (f) conductivity of Spiro-OMeTAD.

Table. S8 Summary of electrochemical data of the HTMs used in this study

**HTM**	E_{ox} / eV (vs Fc / Fc^+)	E_{re} / eV (vs Fc / Fc^+)	$E_{orp}^{\frac{1}{2}}$ / eV (vs Fc / Fc^+)	HOMO / eV (vs NHE)
pcz-SM-DM	0.12	0.01	0.06	-5.10
pcz-DM	0.09	0.00	0.04	-5.08
pcz-SM	0.15	0.04	0.08	-5.12
Spiro-OMeTAD	0.06	0.01	0.03	-5.07

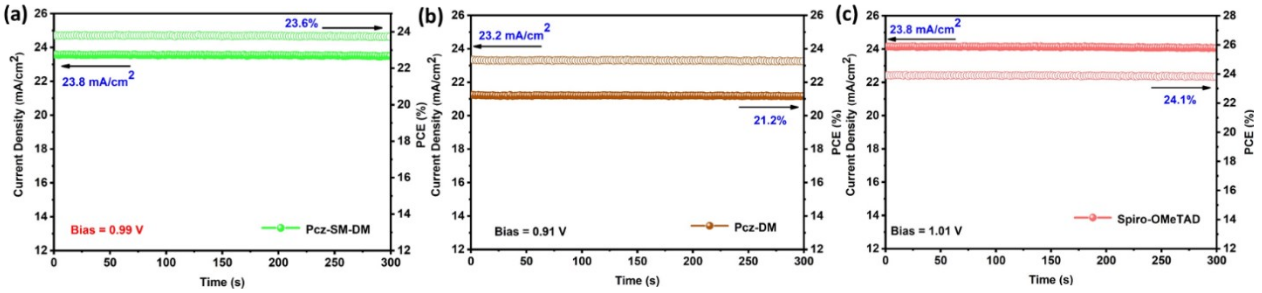

Figure. S18 Steady current density and PCE at maximum power point.

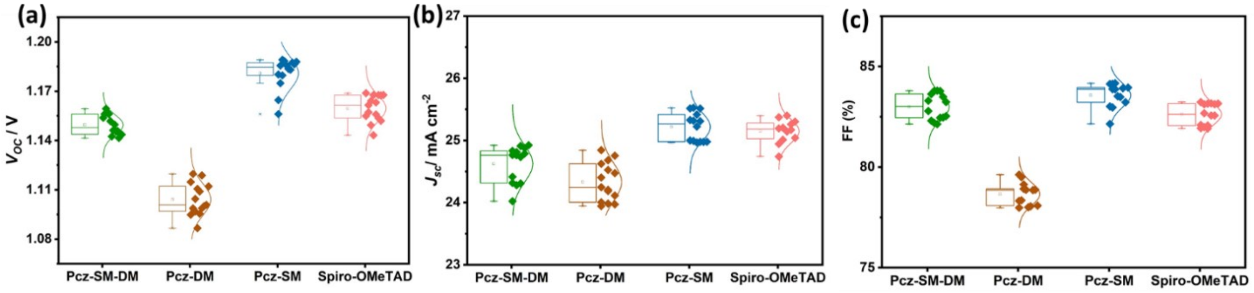

Figure. S19 (a) V_{oc} (b) J_{sc} and (c) FF distribution statistics of pcz-SM-DM, pcz-DM, pcz-SM, and Spiro-OMeTAD as HTMs.

Table S9. Fitted parameters of photoluminescence decay curves of perovskite and HTMs /perovskite.

Sample	A1	τ1 (ns)	A2	τ2 (ns)	τ (ns)
Perovskite	7827.0	101.3	1936.0	1220	940.5
Pcz-DM	8201.4	97.1	1910.0	948.0	687.9
Pcz-SM-DM	10455.5	73.4	1862.9	855.4	601.9
Pcz-SM	32069.5	50.9	1245.5	778.7	319.7
Spiro-OMeTAD	17270.3	61.4	1911.3	911.9	590.5

Reference

1. *Cheng, C., et al., Highly Efficient Phenoxazine Core Unit Based Hole Transport Materials for Hysteresis-Free Perovskite Solar Cells*. ACS Appl. Mater. Interfaces 2018, **10**, 36608−36614
2. *Biyi , W., et al., Constructing Efficient Hole-Transporting Materials by Tuning Fluorine Substitution for Inverted Perovskite Solar Cells with Efficiency Exceeding 20%.* ACS Applied Energy Materials 2022 5 **(5),** 5901-5908
3. *Ravi, M., et al., Asymmetrical benzothiadiazole core-based hole transport materials for planar perovskite solar cell*. Solar Energy Materials and Solar Cells, 2024. **272**: p. 112914.
4. *Xia, Z., et al., Dimeric Carbazole Core Based Dopant-Free Hole Transport Material for n-i-p Planar Perovskite Solar Cell. Advanced Functional Materials, 2024. 34(48): p. 2408423.*
